# Early microvascular coronary endothelial dysfunction precedes pembrolizumab-induced cardiotoxicity. Preventive role of high dose of atorvastatin

**DOI:** 10.1007/s00395-024-01046-0

**Published:** 2024-03-23

**Authors:** Panagiotis Efentakis, Angeliki Choustoulaki, Grzegorz Kwiatkowski, Aimilia Varela, Ioannis V. Kostopoulos, George Tsekenis, Ioannis Ntanasis-Stathopoulos, Anastasios Georgoulis, Constantinos E. Vorgias, Harikleia Gakiopoulou, Alexandros Briasoulis, Constantinos H. Davos, Nikolaos Kostomitsopoulos, Ourania Tsitsilonis, Meletios Athanasios Dimopoulos, Evangelos Terpos, Stefan Chłopicki, Maria Gavriatopoulou, Ioanna Andreadou

**Affiliations:** 1 Laboratory of Pharmacology, Faculty of Pharmacy, National and Kapodistrian University of Athens, Panepistimiopolis, Zografou, 15771 Athens, Greece; 2 Jagiellonian Centre for Experimental Therapeutics (JCET), Jagiellonian University, Kraków, Poland; 3 Biomedical Research Foundation of the Academy of Athens, Athens, Greece; 4 Flow Cytometry Unit, Section of Animal and Human Physiology, Department of Biology, National and Kapodistrian University of Athens, Athens, Greece; 5 Department of Clinical Therapeutics, School of Medicine, National and Kapodistrian University of Athens, Athens, Greece; 6 Department of Biochemistry & Molecular Biology, Faculty of Biology, National and Kapodistrian University of Athens, Athens, Greece; 7 Department of Pathology, School of Medicine, National and Kapodistrian University of Athens, Athens, Greece; 8 Medical College, Jagiellonian University, Krakow, Poland

**Keywords:** Immune checkpoint inhibitors, Pembrolizumab, Microvascular coronary endothelial dysfunction, Cardiotoxicity, Atorvastatin, Cardio-oncology

## Abstract

Immune checkpoint inhibitors (ICIs) exhibit remarkable antitumor activity and immune-related cardiotoxicity of unknown pathomechanism. The aim of the study was to investigate the ICI class-dependent cardiotoxicity in vitro and pembrolizumab’s (Pem’s) cardiotoxicity in vivo, seeking for translational prevention means. Cytotoxicity was investigated in primary cardiomyocytes and splenocytes, incubated with ipilimumab, Pem and avelumab. Pem’s cross-reactivity was assessed by circular dichroism (CD) on biotechnologically produced human and murine PD-1 and in silico. C57BL6/J male mice received IgG4 or Pem for 2 and 5 weeks. Echocardiography, histology, and molecular analyses were performed. Coronary blood flow velocity mapping and cardiac magnetic resonance imaging were conducted at 2 weeks. Human EA.hy926 endothelial cells were incubated with Pem-conditioned media from human mononuclear cells, in presence and absence of statins and viability and molecular signaling were assessed. Atorvastatin (20 mg/kg, daily) was administered in vivo*,* as prophylaxis. Only Pem exerted immune-related cytotoxicity in vitro. Pem’s cross-reactivity with the murine PD-1 was confirmed by CD and docking. In vivo, Pem initiated coronary endothelial and diastolic dysfunction at 2 weeks and systolic dysfunction at 5 weeks. At 2 weeks, Pem induced ICAM-1 and iNOS expression and intracardiac leukocyte infiltration. At 5 weeks, Pem exacerbated endothelial activation and triggered cardiac inflammation. Pem led to immune-related cytotoxicity in EA.hy926 cells, which was prevented by atorvastatin. Atorvastatin mitigated functional deficits, by inhibiting endothelial dysfunction in vivo. We established for the first time an in vivo model of Pem-induced cardiotoxicity. Coronary endothelial dysfunction precedes Pem-induced cardiotoxicity, whereas atorvastatin emerges as a novel prophylactic therapy.

**Supplementary Information:**

The online version contains supplementary material available at 10.1007/s00395-024-01046-0.

## Introduction

Immune checkpoint inhibitors (ICIs) have revolutionized cancer therapy over the last decade [43], since the approval of the first ICI, ipilimumab, in 2011 [6]. Currently, antibodies targeting four immune checkpoints, namely cytotoxic T lymphocyte-associated antigen 4 (CTLA-4), programmed death 1 (PD-1) and its ligand (PD-L1), and lymphocyte activation gene 3 (LAG-3), are approved by the United States and European regulatory authorities as anticancer agents, either as monotherapy or as adjuvant therapy. Near 50% of all patients with metastatic malignancies are under ICI therapy [25]. ICIs maintain a long-lasting antitumor potential, whereas their combination therapies present increased efficacy [58]. Nevertheless, ICI-induced immune-related adverse events are observed. These are triggered by the dysregulation of T-cell immunologic self-tolerance, which might affect multiple organs, including the myocardium [21, 58, 82]. Although infrequent, cardiotoxicity may be life-threatening. The molecular basis of these immune-related adverse events remains marginally understood, but immune mechanisms are highly implicated [80].

Current European Society of Cardiology (ESC) Cardio-Oncology guidelines emphasize the cardiovascular complications of anticancer therapies. Cardiovascular diseases and cancer share common confounders and seem to be cross-linked through cardiovascular toxicities [30]. Regarding ICI-related cardiovascular adverse events (CVAEs), there is an unmet clinical need for efficient management [48]. The largest observational, retrospective, pharmacovigilance study of 122 patients with ICI-associated myocarditis presented an early onset of symptoms, which resulted in 50% mortality in affected patients [72]. Long-term CVAEs (> 90 days) are less well-characterized but are generally manifested in the form of noninflammatory heart failure (HF), accelerated atherosclerosis, and hypertension, resulting in increased mortality rates [14]. Prompt diagnosis and initiation of high-dose corticosteroids within 24 h are important mitigation strategies to improve the outcomes of affected patients [86]. However, due to the shortage of evidence-based recommendations, the monitoring and management of ICI therapy-related CVAEs remain elusive [48].

Up-to-date, all established in vivo models of anti-PD-1 cardiotoxicity have used anti-PD-1 antibodies that are not clinically applicable, but are reactive specifically with the murine PD-1 [22, 54, 84, 87]. Besides the limitation in translation, regarding the use of nonclinically relevant anti-PD-1 antibodies, in vivo studies have also employed an aggressive dose regimen, that of 200 μg/animal, correlating to a human equivalent dose of 8 mg/kg, which is 4 times higher and near 2 times higher than the approved dose for pembrolizumab (Pem) and nivolumab, respectively [23]. Consequently, basic science lacks appropriate preclinical models, to investigate ICI-induced CVAEs. Moreover, despite the fact that some underlying cardiotoxicity mechanisms have been proposed, including the imputation of ICI-related endothelial dysfunction [54], the exact pathomechanism of ICI-induced cardiotoxicity remains elusive.

The scope of the current study was to establish a translational approach, elucidating ICI-induced cardiotoxicity, seeking to **i)** investigate the drug or class effect of ICIs on cardiac homeostasis, **ii)** establish translational in vitro and in vivo models, with clinically used ICIs, by performing in vitro experiments on isolated primary adult murine cardiomyocytes (pAVCs) and splenocytes and in vivo experiments, implementing state-of-the-art functional analyses such as echocardiography, cardiac magnetic resonance imaging (cMRI) and Doppler coronary blood flow velocity (BFV) mapping, on nontumor-bearing mice, **iii)** verify the cross-reactivity of Pem with the respective murine epitopes, by biotechnological production of both mouse and human epitopes and circular dichroism (CD) and in silico analyses, with the human epitopes to be used as a positive binding control and to confirm it in vivo by flow cytometry experiments, **iv)** scrutinize the underlying molecular mechanisms of ICI-induced cardiotoxicity, in a time- and dose-dependent manner and establish strong causal relations between molecular signaling and the observed phenotype, by immunoblotting, confocal microscopy and flow cytometry experiments, **v)** confirm the mechanistic findings in human cell-based in vitro studies on human peripheral mononuclear cells (PBMCs) and human endothelial EA.hy926 cells and **vi)** discover an evidence-based translational therapy against ICI-induced cardiotoxicity, which shall not hamper their antitumor potency, but concomitantly prevent cardiovascular complications.

## Methods

For complete methods, please refer to the supplemental materials online.

### Animals

One hundred sixty male C57Bl/6 J mice, 12–14 weeks of age, were used for conducting this study (**Supplemental **Fig. 1). Experiments were performed in accordance with the “Guide for the Care and Use of Laboratory Animals” and experiments were approved by the Greek and Polish ethics committees (approval number: 166542–01/03/21 and #45/2023, respectively). Animals were housed and maintained in specific pathogen-free cages (8/cage; 25 ± 1 °C) at least for one week before the experiments, according to animal research reporting of in vivo experiments (ARRIVE) guidelines [38]. For the in vitro experiments, twelve animals were sacrificed for the isolation of pAVCs and splenocytes. For the in vivo protocols, mice were randomized as follows: **i.** control (IgG4) group: receiving human IgG4, kappa isotype control (#ab288148, Abcam, Cambridge, UK) (*n* = 9) and **ii.** Pem group: receiving Pem (KEYTRUDA® 25 mg/ml, Merck & Co., Inc., Rahway, USA) (*n* = 9). Antibodies were administered at a dose of 2 mg/kg, weekly, intraperitoneally for five weeks. In a second experimental series and due to the identification of early cardiotoxicity of Pem, experiments were repeated for two weeks (*n* = 5/group) for tissue sampling. In a third and fourth experimental series, experiments were repeated up to two weeks for the conduction of coronary BFV mapping (*n* = 6/group) and cMRI (*n* = 6/group). In a fifth and sixth experimental series, atorvastatin (Atorv) (20 mg/kg) was administered daily via oral gavage as a potent prophylactic therapy for two weeks, concomitantly with IgG4 and Pem treatment (**Supplemental **Fig. 1) and mice underwent BFV mapping (*n* = 6/group) and cMRI (*n* = 6/group) and were subsequently sacrificed for tissue and blood sampling. In a seventh experimental series, the Atorv cohort was repeated for 5 weeks (*n* = 6/group), and mice underwent echocardiography and molecular analysis at the endpoint.

**Fig. 1:**
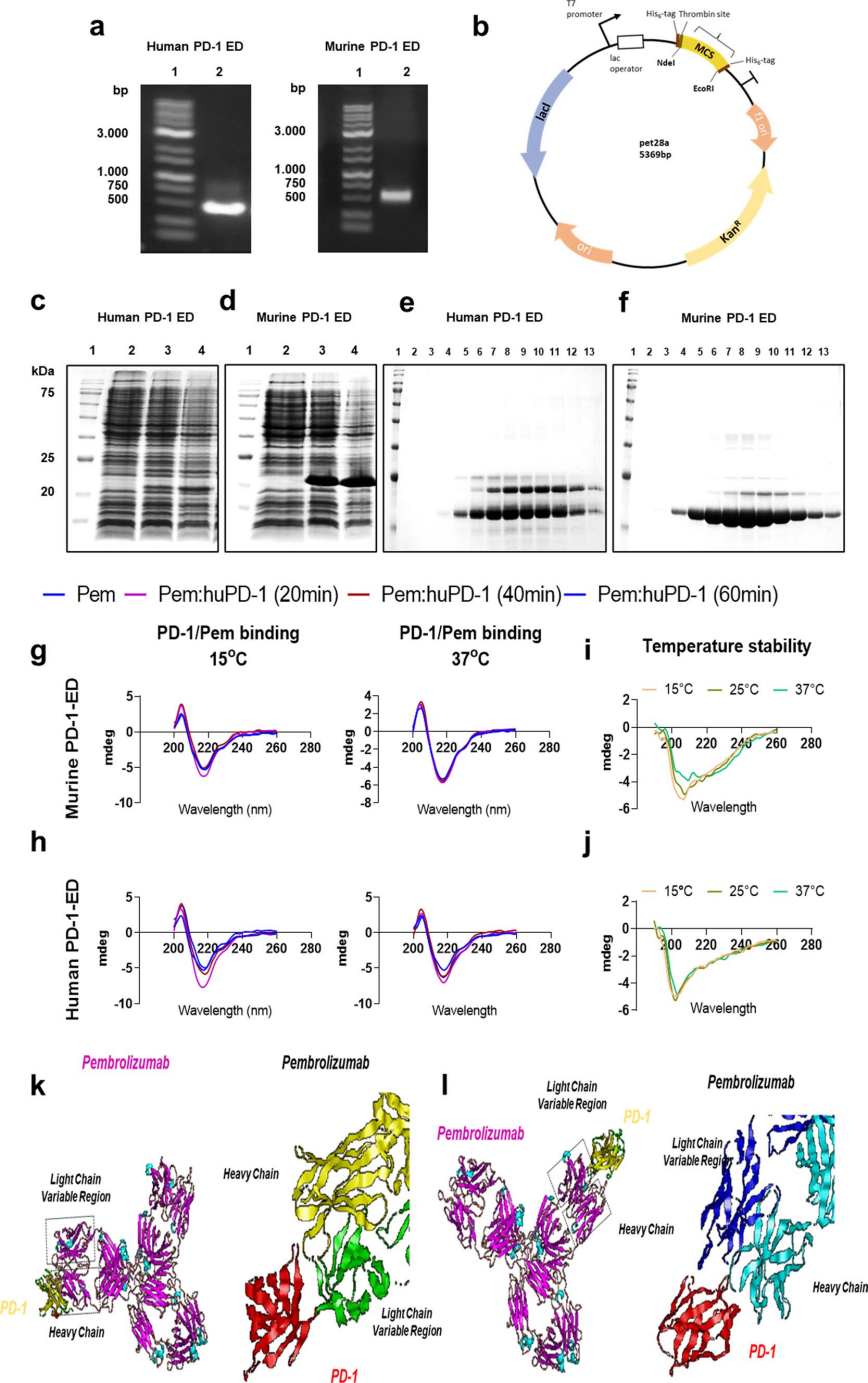
Biotechnological production of human and murine PD-1-ED. Confirmation of pembrolizumab’s cross-reactivity. **a** Agarose gels of PCR-amplified cDNA fragment encoding the human and murine PD-1-ED.** b** Plasmid map of pet28a vector depicting the major plasmid genes and the multiple cloning site. SDS-PAGE of total proteins expressed and extracted from *E. coli* expression cells overexpressing** c** human and** d** murine PD-1-EDs. SDS-PAGE of the purification of** e** human PD-1 and **f.** murine PD-1-EDs with gradient elution. Representative circular dichroism (CD) graphs of pembrolizumab binding with the **g.** murine and **h** human PD-1-ED at 15 °C and 37 °C. Representative CD graphs of** i** murine and** j** human PD-1-ED conformational stability at 15–37 °C. **k-l** Lowest energy conformational models of Pem and murine PD-1-ED binding in silico. *ED* extracellular domain, *PD-1* programmed death 1, *Pem* pembrolizumab

### Dose selection

The dose selection was performed according to currently approved recommendations for the Pem dose regimen in patients with melanoma and with non–small cell lung cancer (2 mg/kg every 3 weeks) [23]. The weekly administration of Pem in mice was chosen due to the relatively high metabolic and renal filtration turnover of the mice compared to humans [64]. The maximal duration of 5 weeks was selected according to ARRIVE animal welfare criteria [38], in line with the previously established in vivo model of anti-PD-1-induced cardiotoxicity [22, 54, 84, 87] and based on our weekly echocardiography analysis. Administrations were performed up to the point that that human IgG4 isotype control antibody did not deteriorate systolic cardiac function, due to cross-species autoimmune reactions. No interspecies dose extrapolation between mice and humans was performed for the antibodies, in compliance with pharmacology guidelines referring to the use of therapeutic antibodies in murine models [75]. The selection of this dose regimen and the use of Pem herein pivotally increased the translational value of our study, since all the up-to-now conducted preclinical studies employ mouse-reactive, nonclinically used anti-PD-1 antibodies and rely on high doses of the antibody (8 mg/kg) to induce the cardiotoxic phenotype [22, 51, 54, 84]. Atorv dose regimen was selected according to previous cardio-oncology preclinical studies, investigating its prophylactic potential against doxorubicin-induced cardiotoxicity in vivo [20, 61], which corresponds to a human equivalent dose of 80 mg [75]. The selected dose translates to the highest approved human dose of 80 mg, according to interspecies dose conversion formulas, taking into account the interspecies differences concerning the pharmacokinetics (metabolic rate, body surface, and volume of distribution) between humans and mice [60]. The selection of the high dose versus low dose of Atorv was also based on our in vitro studies on human PBMCs and endothelial EAhy.926 cells, in which only the high concentration of Atorv exerted cytoprotection against Pem-induced cytotoxicity.

### Immune checkpoint inhibitors and chemicals

The clinically applicable immune checkpoint inhibitors Pem (anti-PD-1 antibody, Pem, KEYTRUDA® 25 mg/ml, Merck & Co., Inc., Rahway, USA), ipilimumab (anti-CTLA-4 antibody, Ipi, 5 mg/ml, YERVOY®, Bristol‑Myers Squibb, NJ, USA), and avelumab (anti-PD-L1 antibody, Ave, BAVENCIO®, 20 mg/ml, Merck & Co., Inc., Rahway, USA) were used in this study. ICIs were kindly donated by the Department of Clinical Therapeutics, School of Medicine, National and Kapodistrian University of Athens. All other chemicals were purchased by Sigma Aldrich (Missouri, USA), unless otherwise stated.

### Human in vitro studies

Ten volunteers kindly donated whole blood for PBMC isolation and one volunteer for cloning of the extracellular domain (ED) of the human *PDCD1* gene. Studies were conducted in compliance with the human studies committee of “Alexandra” General Hospital, Department of Clinical Therapeutics, School of Medicine, National and Kapodistrian University of Athens (#216/ 16–3-2023), the FDA and EMA guidelines and 1964 Declaration of Helsinki and its later amendments. Volunteers signed a written informed consent. Identifying information, including volunteers’ names and initials, was omitted due to the general data protection regulation. PBMCs were further processed as described in the manuscript.

### Statistical Analysis

Data are presented as means ± standard deviation (SD). Continuous variables were compared between two groups using parametric, unpaired student’s *t*-test without assumption of consistent means and among more than two groups using one-way analysis of variance (ANOVA) with Tukey’s post hoc comparisons. Two-way ANOVA was used in the time-course assessment of Pem-induced systolic dysfunction, cytokine profiling using multiplex analyses, and time-course monitoring of circulating cardiac damage biomarkers, and Tukey’s post hoc comparisons were performed. No assumption of equal variability of differences was performed, and data were corrected with Greenhouse–Geisser correction. A *P*-value of at least < 0.05 was considered statistically significant. All statistical analyses and graph preparation were performed using GraphPad Prism 8.5 analysis software (GraphPad Software, Inc., La Jolla, CA, USA). No outliers due to biological diversity were excluded. Samples that did not meet our technical criteria were not included in the analyses a priori. The absence of outlying values was confirmed by GraphPad Prism analysis software, using the ROUT method and Q = 1%.

## Results

### Pembrolizumab induces a Th17-type phenotype in primary splenocytes and an immune cell-mediated cytotoxicity in pAVCs, treated with splenocytes pembrolizumab-conditioned media

Initially, we sought to establish an in vitro model of ICI-induced cardiotoxicity and to decipher whether the observed cardiotoxicity is class- and dose-dependent, as well as whether it is of immune origin or if it is directly induced on the primary cardiomyocytes. In order to investigate the effect of the drugs on the immune cell (IC) compartment in mice, primary splenocytes were treated with one antibody of each class of ICIs, namely Pem, Ipi, and Ave, used at clinically relevant concentrations (0–100 μg/ml) (**Supplemental **Fig. 1a) [17, 32, 35]. Pem and Ave did not lead to any cytotoxicity in the splenocytes (**Supplemental **Fig. 2a), whereas Pem at the high doses led to a generalized inflammatory response and increased the Th17-type cytokines mRNA expression, namely *Tnfa, Il6, Il10, Il17α, *and* Ifnγ* [74]. Ave at the high doses led only to upregulation of *Tnfa*, *Ifnγ,* and *Padi4* mRNA expression, with the latter being a surrogate marker of netosis (**Supplemental **Fig. 2b) [68]. The aforementioned findings are possibly indicative of the divergent inflammatory pathways induced by the anti-PD-1 and anti-PD-L1 antibodies. Interestingly, Ipi at 12.5–100 μg/ml led to primary splenocytes’ cytotoxicity, while this cytotoxicity was not associated with the induction of inflammation (**Supplemental **Fig. 2a–b). This effect might be associated with the IgG1 class of Ipi [69] or with off-target phenomena of the antibody on murine splenocytes and does not correlate with Th17 cell activation that is observed in the clinical setting [18], as only *Il17α* mRNA expression was increased in the absence of *Ifnγ* overexpression (**Supplemental **Fig. 2b) [57]. Therefore, Ipi was excluded from subsequent experiments. Thereupon, we investigated whether Pem and Ave can induce direct toxicity on pAVCs; however, no direct impairment of pAVCs’ viability was observed by these ICIs (**Supplemental **Fig. 2c**)**. On the contrary, transfer of splenocytes’ conditioned media, previously treated for 24 h with Pem, onto pAVCs, led to pAVCs toxicity at 50 and 100 μg/ml, while Ave-conditioned media did not show any toxicity (**Supplemental **Fig. 2d**)**. Pem’s IC-mediated cytotoxicity was accompanied by the induction of inflammation, as shown by the increased *Il6*, *Tnfa*, *Tgf-β*, *Il8* and *Rela* mRNA expression favorably at 100 μg/ml, and autophagy and ER stress both at 50 and 100 μg/ml as shown by *Lc3b/a*, *Atg5* and *Canx, Ddit3,* respectively (**Supplemental **Fig. 2e–f**)**.

**Fig. 2:**
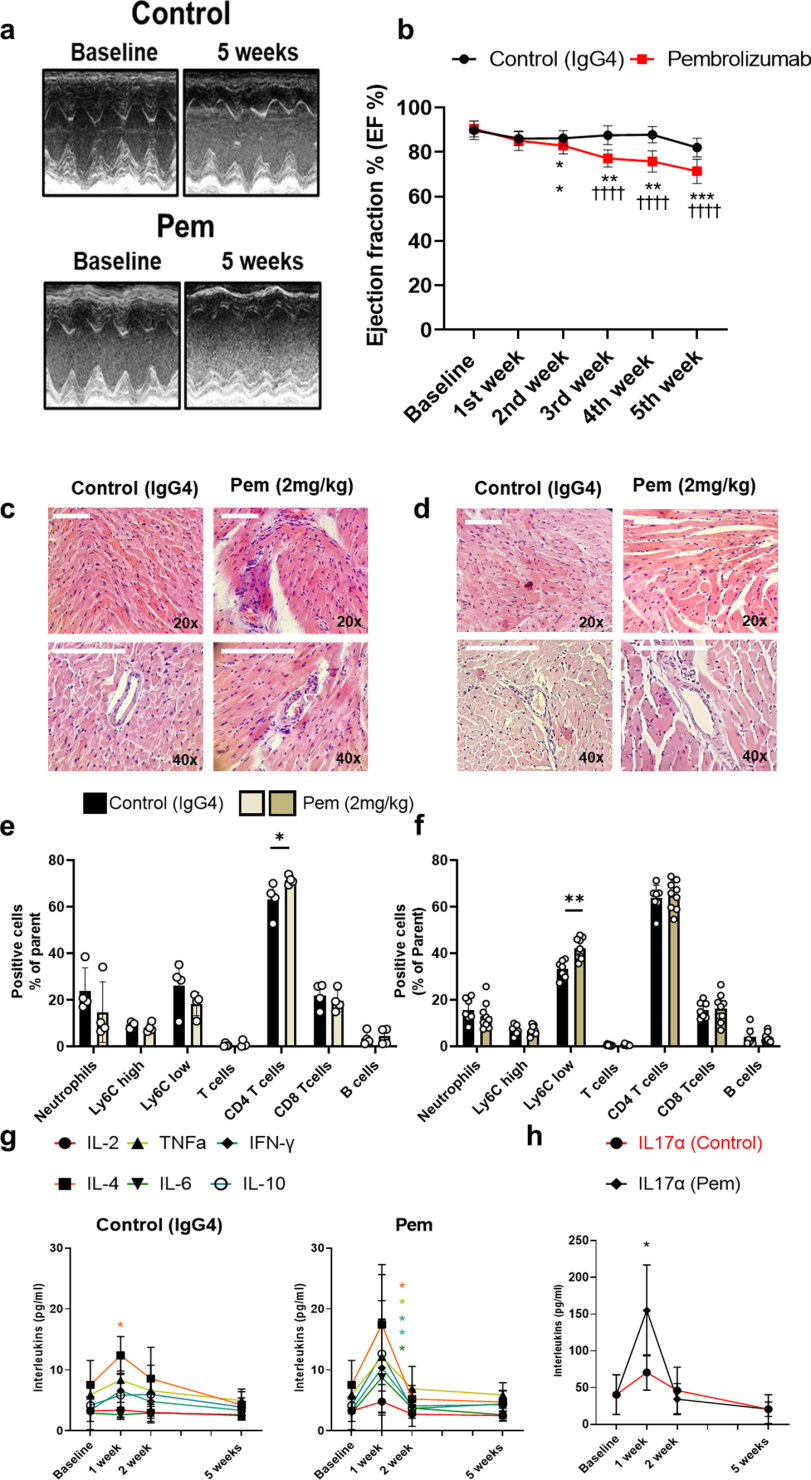
Pembrolizumab leads to a progressive cardiac dysfunction, intracardiac immune cell infiltration, and acute Th17-type cytokine storm.** a** Representative M-mode images from 5 weeks and **b** time-course graph of % ejection fraction in the IgG4-treated controls and Pem-treated mice (*n* = 9/group). Representative hematoxylin–eosin histology images (white bar corresponds to 100 μm, scale shown on images) from the **c** 2- and **d** 5-week groups. Graph of the flow cytometry subpopulation analysis of the myocardial tissue at **e** 2- and **f** 5-week cohorts. Time course graphs of the multiplex analysis of circulatory **g** Th17-type cytokines (pg/ml) and **h** IL-17α (pg/ml) in the IgG4-treated controls and Pem-treated mice (*n* = 6/group). Data are presented as mean ± SD. *P < 0.05,**P < 0.01 vs controls, ††††*P* < 0.001 vs Baseline. Unpaired Student’s *t*-test or two-way ANOVA of variance, Tukey’s post hoc analysis. *IFN-γ* interferon gamma, *IL* interleukin, *Pem* pembrolizumab, *TNF-α* tumor necrosis factor alpha

### Pembrolizumab binds to the murine PD-1-ED, similar to the human PD-1-ED, in a conformational-dependent manner

Based on our in vitro model of ICI-induced cardiotoxicity and our results on Pem-induced Th17-type activation in splenocytes (**Supplemental **Fig. 2), we sought to verify Pem’s binding with the murine PD-1-ED in vitro, using human PD-1-ED as a positive binding control, both biotechnologically produced in an *E. coli* system (Fig. 1a–f). The recombinant PD-1-EDs presented similar CD spectra at 15 °C, whereas secondary structure content % was similar for the two protein equivalents, as estimated by BeStSel analysis (**Supplemental Table 1**) [56]. Regarding Pem’s interaction with the murine and human PD-1-ED, Pem incubation with both PD-1-EDs induced the same structural rearrangements to the antibody’s secondary structure, thus confirming its binding to both recombinant proteins at 15 °C (Fig. 1g–h). Nevertheless, in the case of the murine PD-1-ED, CD spectral shifts were evident only at 15 °C, while binding between its human counterpart and Pem was also observed at 37 °C, indicating that the affinity of the antibody for the murine epitope is temperature- and conformation-dependent. A comparison of the CD spectra, acquired for the murine and human PD-1-ED, confirmed the reduced stability of the murine epitope at 37 °C (Fig. 1i–j**)**. The specificity of Pem’s binding to PD-1-ED was confirmed using IgG4 as a negative control, which showed no structural rearrangements upon interaction with either the human or the murine PD-1-ED (**Supplemental **Fig. 3). In silico protein–protein docking confirmed the binding of Pem with the murine PD-1-ED between the heavy and light chains of Pem, which was similar to what was already shown for the Pem and human PD-1-ED binding in literature [66] and in line with the similar secondary structure content % previously shown for the two protein equivalents (**Supplemental Table 1, **Fig. 1k–l**)**. Taking into account **i.** the induction of Th17-type phenotype in primary splenocytes (**Supplemental **Fig. 2**)** and **ii.** the novel confirmation of Pem binding to the murine epitope, we subsequently sought to establish an in vivo cardiotoxicity model.

**Fig. 3:**
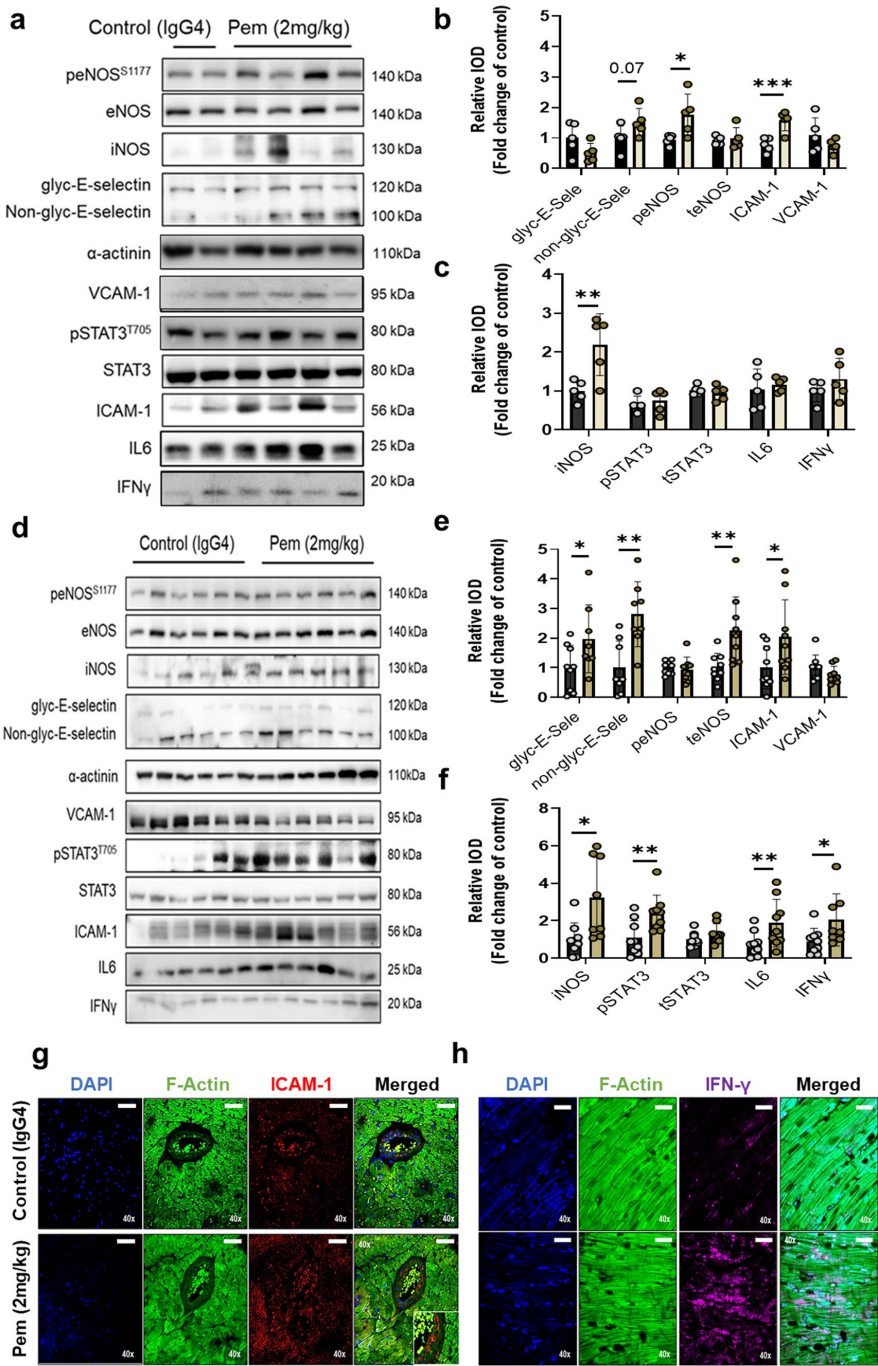
Pembrolizumab leads to early and progressive coronary endothelial inflammatory activation.** a** Representative Western blot images and relative densitometry analysis of **b** endothelial and **c** inflammation markers at 2 weeks (*n* = 5/group). **d** Representative Western blot images and relative densitometry analysis of **e** endothelial and **f** inflammation markers at 5 weeks (*n* = 9/group). Representative confocal microscopy images of DAPI (blue), F-actin (green), and **g** ICAM-1 (red) or **h** IFN-γ (magenta) in the myocardium at 5 weeks (bar corresponds to 100 μm; scale is shown on images). Data are presented as mean ± SD. *P < 0.05,**P < 0.01, ***P < 0.005 vs controls. Unpaired Student’s *t*-test. *eNOS* endothelial nitric oxide synthase*, ICAM-1* intercellular adhesion molecule 1*, IFN-γ* interferon gamma*, IL-6* interleukin 6*, iNOS* inducible nitric oxide synthase*, STAT3* signal transducer and activator of transcription 3*, VCAM-1* vascular cell adhesion molecule 1

### Pembrolizumab leads to transient T helper cell infiltration and a progressive decline in cardiac function

Based on the translational dose regimens, we subsequently established the in vivo murine model of Pem-induced cardiotoxicity (**Supplemental **Fig. 1b). Weekly Pem administration led to an early systolic dysfunction, evident as a significant decline in ejection fraction % (EF %) compared to baseline at 2 weeks. The impairment of systolic function was further exacerbated at 5 weeks of treatment, compared to IgG4-treated controls and baseline (Fig. 2a–b**, Supplemental Table 2–3**). Histological evaluation of the myocardium at 2 and 5 weeks indicated an early intracardiac and perivascular IC infiltration (vasculitis) at 2 weeks (Fig. 2c) and a shrinkage of cardiomyocytes, accompanied by signs of myocytolysis and disruption of the coronary arterial wall at 5 weeks, indicative of exacerbated histological damage (Fig. 2d) [62]. Flow cytometry indicated an early increase in CD4 T helper cells in the myocardium at 2 weeks which shifted to increased Ly6C_low_ macrophages at 5 weeks (Fig. 2e–f**, Supplemental **Fig. 4).

**Fig. 4:**
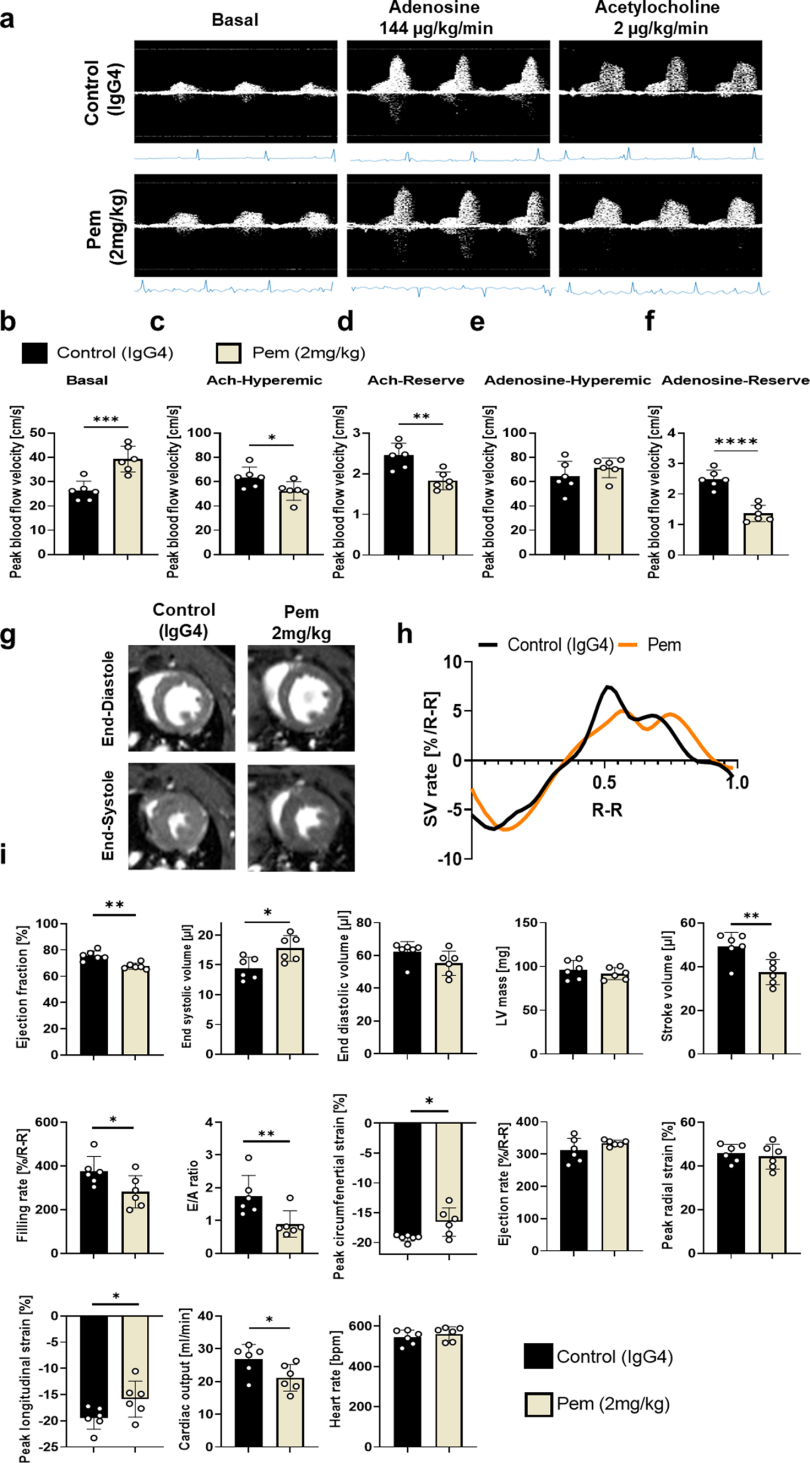
Pembrolizumab induces early systolic and diastolic dysfunction and microvascular endothelial dysfunction. **a–f** Representative images and Doppler blood flow velocity mapping analysis of the IgG4-treated controls and Pem-treated mice (*n* = 6/group) at baseline and after acetylcholine (144 µg/kg/min) and adenosine (144 µg/kg/min) hyperemia. **g–i** Representative cMRI images, SV rate and cMRI analysis of the IgG4-treated controls and Pem-treated mice (*n* = 6/group). Data are presented as mean ± SD. *P < 0.05,**P < 0.01, ***P < 0.005, and **** 0.001 vs controls. Unpaired Student’s *t*-test. *Ach* acetylcholine*, SV* stroke volume

Regarding the circulatory Th17-type cytokine profile, Pem led to the increase in Th17-type cytokines, namely TNF-α, IFN-γ, IL-4, IL-6, and IL-10, except from IL-2, which remained unchanged at 1 week of administration. Notably, the Th17-type cytokines’ increase was rapidly resolved at 2 weeks and remained unchanged until the 5th week of the experiments. Only an increase in IL-4 was observed in IgG4-treated control mice after 1 week of administration. Additionally, a profound increase in IL-17α was observed only in Pem-treated mice, after 1 week of administration (Fig. 2g–h). Therefore, Pem exhibited an early activation of Th17-type cells at 1 week, which led to a mild systolic function impairment and intracardial T helper cell infiltration at 2 weeks, which further proceeded to exacerbated systolic dysfunction, Ly6C_low_ macrophages infiltration, and histologically defined cardiac deficits at 5 weeks. Additionally, we assessed circulatory biomarkers of cardiac damage, namely cardiac troponin I (cTnI), lactate dehydrogenase (LDH) and creatine phosphokinase-MB (CK-MB). We found a significant increase in TnI at 1 week of Pem administration, compared to IgG4-treated controls and a significant TnI increase at 5 weeks of Pem administration, compared to baseline and to controls. LDH was found to be increased at 5 weeks compared to controls, whereas LDH and CK-MB levels did not increase throughout the serial administrations neither in the Pem nor in the IgG4-treated group compared to baseline (**Supplemental **Fig. 5**).**

**Fig. 5:**
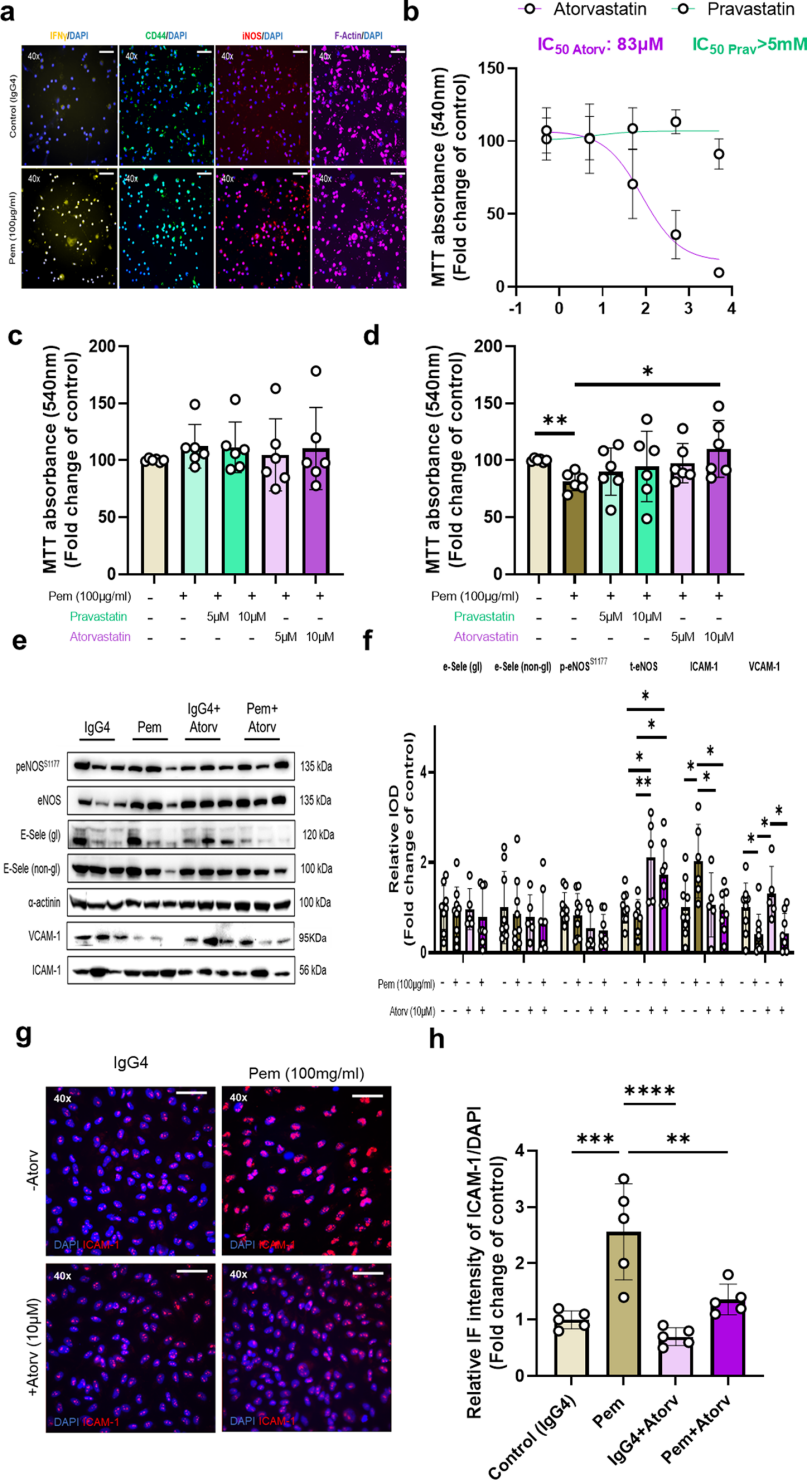
Pembrolizumab induces immune cell-mediated toxicity in human endothelial EA.hy926 cells. Preventive role of high concentration of atorvastatin. **a** Immunofluorescence images of human PBMCs treated with Pem (100 μg/ml) for 72 h stained for IFN-γ, CD44, and iNOS. **b** Graph of cytotoxicity IC_50_ for Prav and Atorv (0.5μΜ–5 mM) (*n* = 6/group) **c** Graph of cellular viability assessed by MTT assay in EA.hy926 endothelial cells treated with PBMC IgG4-conditioned media in the presence of Atorv and Prav (5–10 μM) (*n* = 6/group) and **d** in EA.hy926 endothelial cells treated with PBMC Pem-conditioned media (100 μg/ml) for 24 h (*n* = 6/group) in the presence and absence of Atorv and Prav (5–10 μM). **e** Representative Western Blot images and **f** Relative densitometry analysis of the endothelial markers E-selectin, (phospho-)eNOS, ICAM-1, and VCAM-1 in EA.hy926 endothelial cells treated with PBMC Pem-conditioned media (100 μg/ml) for 24 h (*n* = 6/group) in the presence and absence of Atorv (10 μΜ) (*n* = 5–9/group) **g** Representative immunofluorescent images and** h** relative immunofluorescence intensity normalized to DAPI signal of ICAM-1 in EA.hy926 endothelial cells treated with PBMC Pem-conditioned media (100 μg/ml) for 24 h (*n* = 5/group) in the presence and absence of Atorv. Data are presented as mean ± SD. *P < 0.05, **P < 0.01, ***P < 0.005, ****P < 0.001, one-way ANOVA of variance, and Tukey’s post hoc analysis*. Atorv* atorvastatin, *CD44* cluster differentiation molecule 44*, eNOS* endothelial nitric oxide synthase, *ICAM-1* intracellular adhesion molecule 1*, IFN-γ* interferon gamma*, iNOS* inducible nitric oxide synthase*, PBMCs* peripheral blood mononuclear cells*, Pem* pembrolizumab*, Prav* pravastatin*, VCAM-1* vascular cell adhesion molecule 1

In order to validate our in vitro data, originating from the CD and in silico experiments, we sought to confirm Pem’s cross-reactivity by investigating its effect on T-cell expansion in vivo at 5 weeks. The time point was selected according to clinical data, indicating a Pem-induced T-cell expansion, at least 4 weeks after the initial administration of the drug [36]. We observed an increase in the total T-cell population, characterized by a concomitant increase in CD4^+^ and CD8^+^ cells, without a parallel increase in B-cell or NK-cell populations (**Supplemental **Fig. 6**)**. Taking into account that anti-PD-1 therapy specifically targets CD4^+^ and CD8^+^ T-cell population, without directly affecting B-cell and NK-cells in humans [11, 78], our flow cytometry data reinforce our finding of Pem’s cross-reactivity in the murine model.

**Fig. 6:**
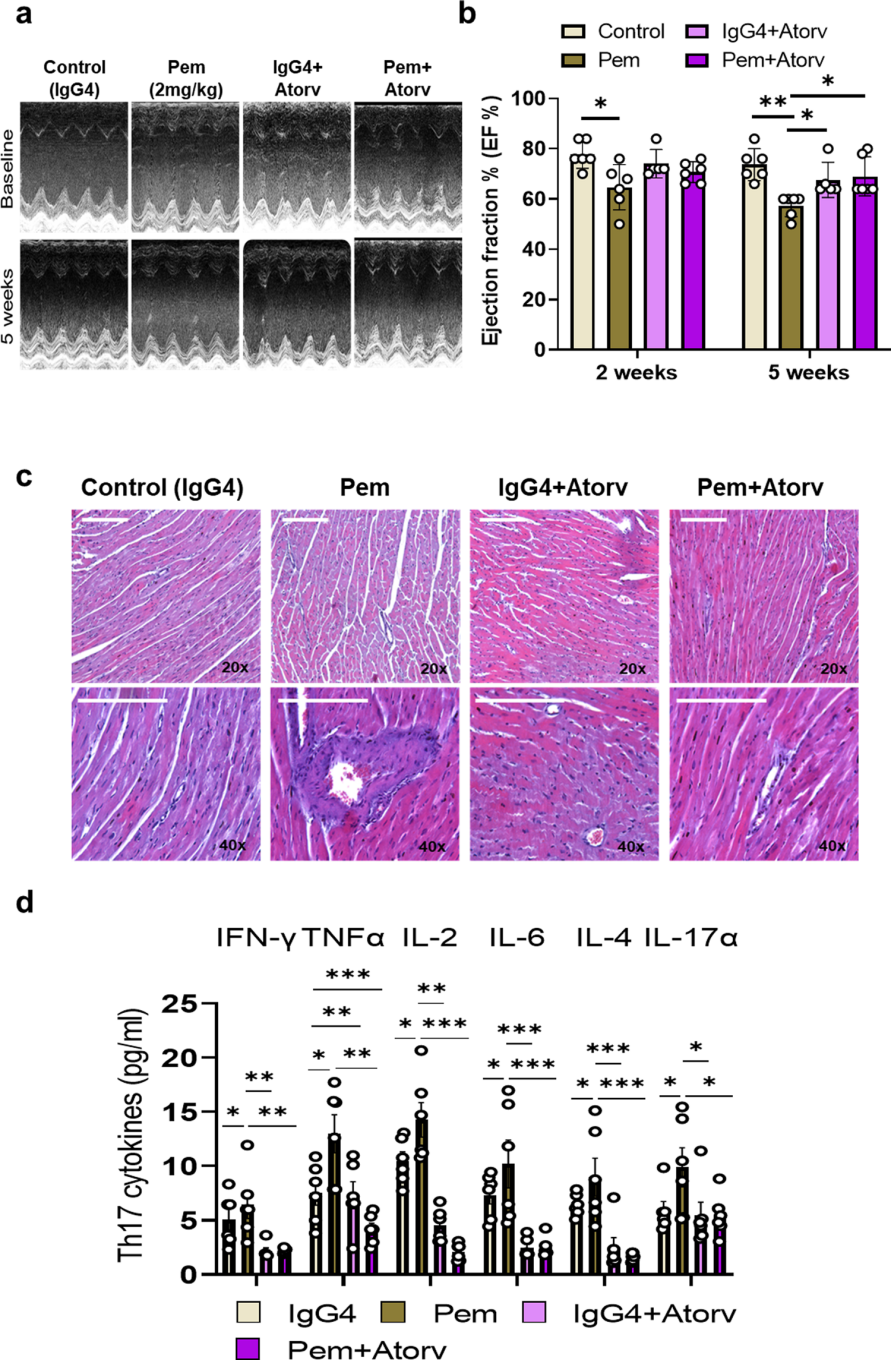
Atorvastatin mitigates early and late cardiac systolic dysfunction, histological deficits, and acute Th17-type cytokines’ storm induced by pembrolizumab. **a** Representative M-mode images at baseline and 5 weeks and **b** graph of % ejection fraction in the IgG4-treated controls and Pem-treated as well as IgG4 + Atorv-treated and Pem + Atorv-treated mice (*n* = 5–6/group). **c** Representative hematoxylin–eosin histology images at 5 weeks of treatment (white bar corresponds to 100 μm; scale is shown on images). **d** Graph of the multiplex analysis of circulatory Th17-type cytokines (pg/ml) at 1 week of administration (*n* = 6/group). Data are presented as mean ± SD. *P < 0.05,**P < 0.01 ***P < 0.005 vs controls, one-way ANOVA of variance, and Tukey’s post hoc analysis. *Atorv* atorvastatin, *IFN-γ* interferon gamma, *IL* interleukin*, Pem* pembrolizumab, *TNF-α* tumor necrosis factor alpha

### Pembrolizumab leads to early and progressive coronary endothelial inflammatory activation

Since Pem-induced acute increase in IL-17α in the circulation and intracardiac IC infiltration suggest coronary endothelial dysfunction [2, 49], we focused our mechanistic studies on endothelial-related pathways and inflammatory mediators in the myocardium. At 2 weeks, Pem administration led to an increase in intercellular adhesion molecule 1 (ICAM-1) and inducible NO synthase (iNOS) and a possibly compensatory increase in endothelial NO synthase (eNOS) phosphorylation (Fig. 3a–c). At 5 weeks, Pem administration led to further endothelial activation, as shown by the significant E-selectin, ICAM-1, and eNOS upregulation (Fig. 3d–e) and a concomitant increase in the inflammatory markers iNOS, IL-6, IFN-γ, and signal transducer and activator of transcription 3 (STAT3) phosphorylation (Fig. 3f). The upregulation of ICAM-1 in the intracardiac coronary vessels and IFN-γ in the sarcomeres of the cardiomyocytes was confirmed by confocal microscopy at 5 weeks after Pem administration (Fig. 3g-h).

### Pembrolizumab leads to alterations in coronary endothelial function and early diastolic and systolic cardiac dysfunction

Pem increased basal BFV at 2 weeks, whereas decreased acetylcholine (Ach)-mediated hyperemic BFV was observed, without changes in adenosine-mediated hyperemic BFV. Both Ach- and adenosine-mediated BFV reserves were decreased in Pem-treated mice at 2 weeks. Therefore, our results strongly confirmed the contribution of microvascular endothelial dysfunction in the early stages of Pem-induced cardiotoxicity, taking under consideration the endothelial-dependent vasodilation induced by Ach (Fig. 4a–f) [41].

Since cMRI is the state-of-art technique for ICIs cardiotoxicity diagnosis [19], we employed the technique in our in vivo model, to obtain a comprehensive analysis of Pem’s cardiotoxicity in terms of systolic and diastolic dysfunction at 2 weeks. Pem led to a significant reduction in EF %, stroke volume, % filling rate, cardiac output, an increase in end-systolic volume and concomitant impairment in % peak longitudinal and circumferential strain, confirming the early systolic dysfunction as previously identified by echocardiography. Additionally, Pem led to a significant decrease in E/A ratio, a surrogate marker of diastolic function (Fig. 4g–i). Conclusively, cMRI and Doppler imaging revealed that Pem at 2 weeks led to microvascular coronary dysfunction and to systolic and diastolic dysfunction in vivo.

### Pembrolizumab induces IC-mediated cytotoxicity in human endothelial cells. Protective effect of atorvastatin

To investigate whether Pem-induced endothelial impairment could be recapitulated in vitro, we studied the endothelial damage in EA.hy926 human endothelial cells. PBMC incubation with Pem at 100 μg/ml for 72 h, corresponding to the antibody’s concentration in human plasma after one dose of Pem [17], led to the activation of PBMCs, as indicated by immunofluorescence analyses of IFN-γ, CD44, and iNOS (Fig. 5a**)**. On a translational approach to reveal a preventive strategy and taking under consideration the endothelial-targeted pharmacological action of statins [83], we employed two statins, pravastatin (Prav) and Atorv, with different lipophilicity and intensity profiles (**Supplemental **Fig. 1c**).** Initially, we tested Prav and Atorv cytotoxicity on EA.hy926 cells in order to identify their half maximal inhibitory concentration (IC_50_) concerning cellular viability. We found that Atorv presented an IC_50_ value of 83 μM, while Prav did not show any cytotoxicity at the tested range of concentrations (IC_50_ > 5 mM) (Fig. 5b). Thereupon, we tested the concentrations of 5 and 10 μM [16], according to literature in the presence of non-stimulated PBMCs’ supernatant in order to confirm the absence of cytotoxicity. We observed no significant cytotoxicity of either Prav or Atorv in the tested concentrations (Fig. 5c**)**. Nevertheless, transfer of Pem-activated PBMCs’ supernatant onto EA.hy926 cells for 24 h led to cytotoxicity (Fig. 5d), confirming the Pem-related, IC-mediated endothelial damage in the human in vitro setting. [12]. Only the high concentration of Atorv, prevented IC-mediated endothelial toxicity by Pem, which was accompanied by upregulation of eNOS and downregulation of ICAM-1 in vitro (Fig. 5e–f). The prevention of Pem-induced ICAM-1 increase by Atorv was also confirmed by immunofluorescence microscopy in the EA.hy926 cells (Fig. 5g–h**)**.

### Atorvastatin mitigates progressive cardiac dysfunction, cardiac injury, and acute Th17 cytokine storm induced by pembrolizumab

In order to confirm our in vitro findings on the preventive role of Atorv against Pem-induced endothelial dysfunction, the cardioprotective potential of high-dose Atorv at 20 mg/kg [33] was studied in vivo (**Supplemental **Fig. 1d). Atorv prevented the progressive Pem-induced cardiac systolic dysfunction, as shown by the preserved EF % at 2 and 5 weeks, respectively (Fig. 6a–b**, Supplemental Table 4–5**). Moreover, Atorv abrogated the histological signs of Pem-induced cardiac injury, such as cardiomyocyte shrinkage and vasculitis (Fig. 6c). Concerning the Th17-type cytokine profile at 1 week of administration, Atorv coadministration led to the inhibition of IFN-γ, TNF-α, IL-2, IL-6, IL-4, and IL-17α release in comparison with the Pem group, in line with its already known immunomodulatory effect (Fig. 6d) [37].

### Atorvastatin prevents microvascular coronary endothelial and diastolic and systolic cardiac dysfunction induced by pembrolizumab

**Fig. 7:**
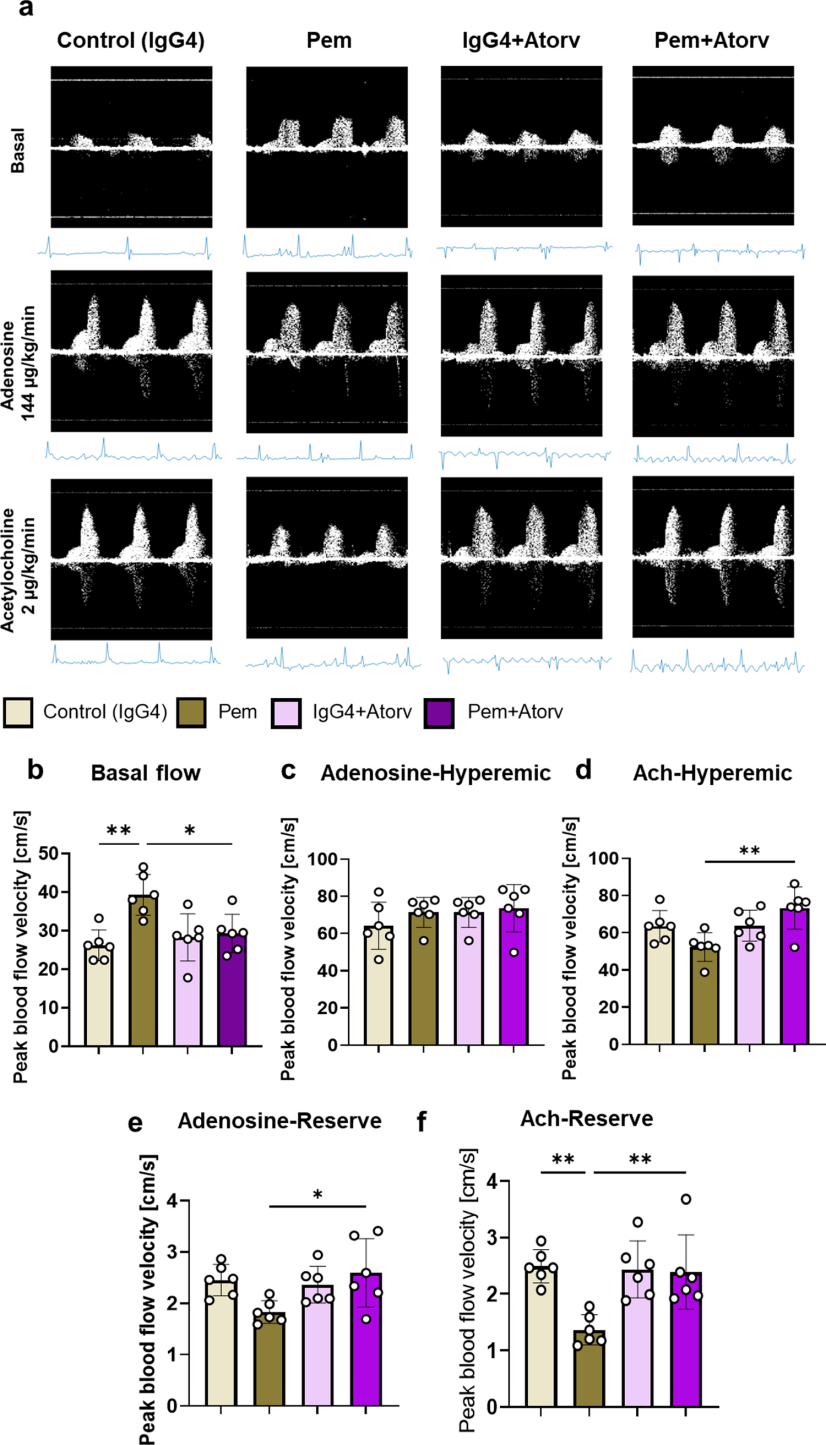
Atorvastatin inhibits early microvascular endothelial dysfunction induced by pembrolizumab. **a** Representative images and **b–f** Doppler blood flow velocity mapping analysis of the IgG4-treated controls and Pem-treated as well as IgG4 + Atorv-treated and Pem + Atorv-treated mice (*n* = 6/group) at baseline and after acetylcholine (144 µg/kg/min) and adenosine (144 µg/kg/min) hyperemia. Data are presented as mean ± SD. *P < 0.05,**P < 0.01 vs controls one-way ANOVA of variance, and Tukey’s post hoc analysis. *Ach* acetylcholine, *Atorv* atorvastatin, *Pem* pembrolizumab

**Fig. 8:**
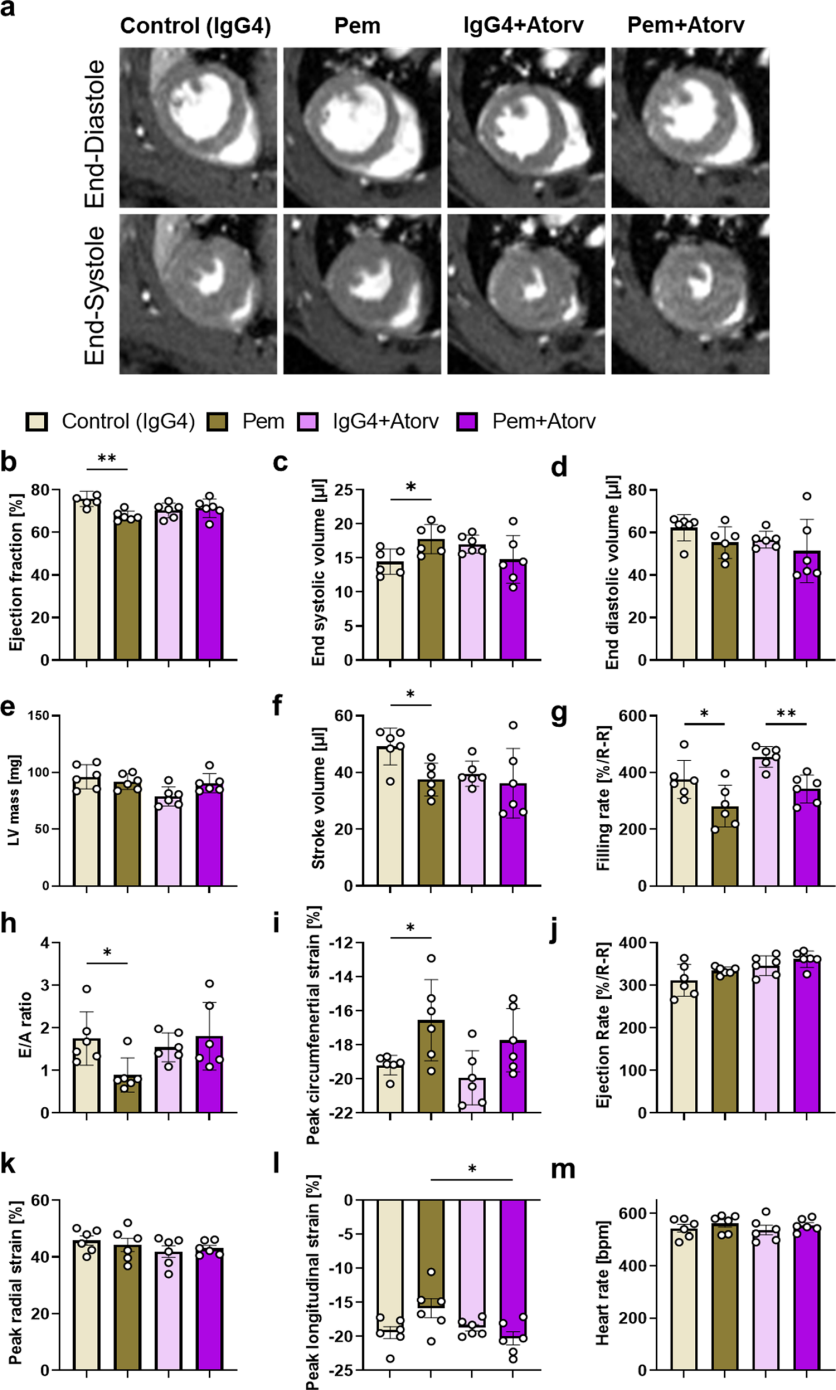
Atorvastatin prevents early cardiac systolic and diastolic dysfunction induced by pembrolizumab. **a** Representative cMRI images at end-diastole and end-systole and **b–m** cMRI analysis concerning cardiac systolic and diastolic function of the IgG4-treated controls and Pem-treated as well as IgG4 + Atorv-treated and Pem + Atorv-treated mice (*n* = 6/group). Data are presented as mean ± SD. *P < 0.05,**P < 0.01 vs controls, one-way ANOVA of variance, and Tukey’s post hoc analysis. *Atorv* Atorvastatin, *LV* left ventricular, *Pem* Pembrolizumab

To confirm that Atorv prophylaxis against Pem-induced cardiotoxicity in vivo was related to effects on microvascular coronary endothelial dysfunction, Atorv was co-administered with Pem up to the 2nd week and cMRI and Doppler coronary BFV mapping were conducted. Atorv coadministration, successfully mitigated Pem-induced basal BFV increase and depressed Ach-mediated hyperemic BFV, without affecting adenosine-mediated hyperemic BFV, revealing a potent microvascular coronary endothelium protection of Atorv against Pem-induced damage at 2 weeks. Atorv mitigated both the decreases in Ach- and adenosine-mediated BFV reserve (Fig. 7). Additionally, Atorv preserved EF %, end-diastolic volume, stroke volume, peak circumferential strain and E/A ratio, which were significantly compromised by Pem. Moreover, it significantly restored peak longitudinal strain compared to Pem group, indicating that an early prophylactic treatment with Atorv prevented the coronary microvascular dysfunction and systolic and diastolic cardiac function impairment induced by Pem (Fig. 8).

### Atorvastatin mitigates coronary endothelial activation and cardiac inflammation induced by Pembrolizumab

**Fig. 9:**
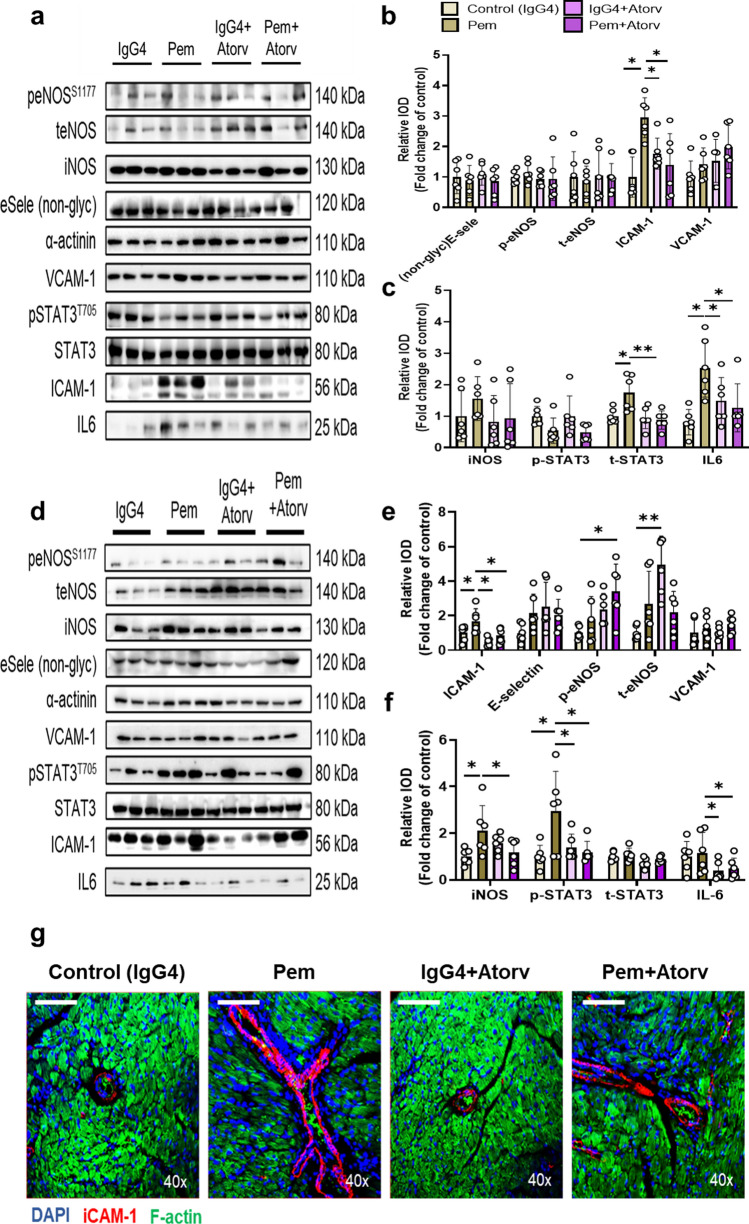
Atorvastatin mitigates early and late endothelial activation and inflammatory signaling upregulated by pembrolizumab. **a** Representative Western blot images and relative densitometry analysis of **b** endothelial and **c** inflammation markers at 2 weeks (*n* = 6/group). **d** Representative Western blot images and relative densitometry analysis of **e** endothelial and **f** inflammation markers at 5 weeks (*n* = 6/group).** g** Representative confocal microscopy images of DAPI (blue), F-actin (green), and ICAM-1 (red) at 5 weeks (bar corresponds to 100 μm, scale shown on images). Data are presented as mean ± SD. *P < 0.05,**P < 0.01, and ***P < 0.005 vs controls. One-way ANOVA of variance and Tukey’s post hoc analysis. *Atorv* atorvastatin, *eNOS* endothelial nitric oxide synthase, *ICAM-1* intercellular adhesion molecule 1*, IL-6* interleukin 6*, iNOS* inducible nitric oxide synthase, *Pem* pembrolizumab, *STAT3* signal transducer and activator of transcription 3, and *VCAM-1* vascular cell adhesion molecule 1

Subsequently, we found that Atorv prevented the increase in ICAM-1, STAT3, and IL-6 at 2 weeks (Fig. 9a–c), whereas it inhibited the upregulation of ICAM-1, iNOS, and STAT3 phosphorylation at 5 weeks in the myocardium. Atorv increased eNOS expression, whereas Atorv coadministration with Pem increased eNOS phosphorylation, compared to IgG4-treated controls. Interestingly, Atorv and its coadministration with Pem reduced myocardial IL-6 expression compared to the Pem group (Fig. 9d–f). The preventive potential of Atorv against Pem-induced ICAM-1 upregulation, at 5 weeks, was also confirmed by confocal microscopy in the intracardiac coronary vessels (Fig. 9g). Conclusively, Atorv acts prophylactically against Pem-cardiotoxicity both at a functional and molecular level.

## Discussion

Herein, we investigated the class and drug effect of ICIs on cardiac function and we have established, for the first time, a translational in vivo model of Pem-induced cardiotoxicity, successfully identifying an evidence-based and clinically applicable prophylactic therapy. Our in vitro findings suggest that among the anti-PD-1, anti-PD-L1 and anti-CTLA-4 antibodies, only Pem exerted notable IC-mediated toxicity in murine pAVCs. The rationale for the use of primary splenocytes relies on the fact that we sought to prove that Pem does not lead to direct cytotoxicity on primary adult cardiomyocytes, while treatment of pAVCs with Pembrolizumab-conditioned media from splenocytes, consisting primarily of immature B and T cells [13], led to significant cytotoxicity in the cardiomyocytes in vitro*,* confirming the IC-mediated nature of Pem’s cardiotoxicity. This finding is in line with clinical observations, as anti-PD-1 therapy presents the highest incidence of CVAEs compared to the other ICIs classes [70]. Since we observed that Pem’s pharmacological effect on primary splenocytes was clinically relevant to the T-cell activation observed in humans [67], we investigated whether the observed effect was on- or off-target.

In vitro studies have previously shown that Pem does not cross-react with the murine PD-1, which up-to-now hindered the establishment of translational in vivo models [44]. However, in all the aforementioned studies, enzyme-linked immunosorbent assays have been employed to test the cross-reactivity of the drug [44]. Herein, we biotechnologically engineered both the human and murine PD-1-EDs, to investigate the cross-reactivity of the antibody in a temperature- and conformational-dependent manner by CD. Initially, we investigated the similarity of the murine and human PD-1-EDs, concerning the estimated secondary structure content %. We found that both proteins have similar secondary structure content %. Taking into account that human PD-1-ED deranges its secondary structure in order to bind to Pem, similar secondary structures of the human and murine PD-1-EDs facilitate the putative cross-reactivity of the antibody [85]. Nevertheless, we observed that murine PD-1-ED undergoes conformational changes at 37 °C, which greatly affects the binding of Pem on the murine epitope. Taking into consideration that enzyme-linked immunosorbent assays often require long incubation time at room temperature or 37 °C [44], our findings on the temperature stability of the protein might explain the reported absence of cross-reactivity of Pem. Previous studies have shown that Pem’s stability and its antigen–antibody binding with the PD-1-ED are affected by stressors such as temperature (≥ 40 °C) [79]. However, the epitope’s temperature stability is not yet investigated and constitutes a novelty of the current study. Therefore, the up-to-now limitations in Pem’s cross-reactivity should be further scrutinized. Additionally, Pem’s binding to murine PD-1-ED, similar to the human PD-1-ED, was confirmed by in silico protein–protein docking experiments, which additionally supported our previous CD findings. Importantly, we sought to confirm Pem’s cross-reactivity in vivo at 5 weeks. We found that in compliance with humans [11, 78], Pem increased the total T-cell count in the whole blood, compared to the IgG4 isotype control, without affecting the B- and NK-cell population. The latter solidifies Pem’s cross-reactivity in our murine model of cardiotoxicity, as Pem induced a clinically relevant immune response in vivo, in line with its pharmacological effect in cancer patients.

Taking into account that **i.** Pem induced a Th17-type cell activation in primary splenocytes, which is similar to the T-cell activation observed in humans [67] and led to IC-mediated cardiotoxicity in the pAVCs, **ii.** human and murine PD-1-EDs presented similar estimated secondary structure content % facilitating the pharmacodynamic interaction of both PD-1-EDs with Pem, **iii.** the similar CD shift of Pem’s spectrum upon incubation with the human and murine PD-1-EDs, **iv.** the in silico confirmation of Pem’s binding with the human and the murine PD-1-EDs originating from low-energy models, and **v.** our in vivo confirmation of Pem-induced T-cell expansion at 5 weeks of administration, we can safely suggest the cross-reactivity of Pem with the murine PD-1-ED, regarding its binding capacity to its ligand PD-L1. These results are of utmost translational significance, as they permit, for the first time, the conduction of in vivo experiments with anti-PD-1 therapeutics, using Pem as a prototype. As for the Pem dosage, the previous preclinical studies have used **i)** anti-PD-1 antibodies that are not clinically applicable and **ii)** 4 times higher doses than the approved dose for Pem. Herein, we established for the first time an in vivo model directly translating the human dose into our murine in vivo model on a bench-to-bedside approach.

Endothelial activation and microvascular coronary endothelial dysfunction were identified as early mediators of Pem’s cardiotoxicity. Early reports indicate that endothelial PD-L1 orchestrates CD8^+^ T-cell-mediated injury in the myocardium, demonstrating an important role of the IFN-γ-inducible PD-L1, in protecting the myocardium against ICIs immune-related adverse events. However, this finding was not further investigated in terms of anti-PD-1 therapy-induced CVAEs [24]. A contemporary study confirmed the expression of PD-L1, mainly in the endothelial compartment of the myocardium and proposed that TNF-α is a key mediator of the early anti-PD-1-related cardiotoxicity [54]. Despite the fact that endothelial dysfunction is part of the early-on anti-PD-1-related cardiotoxicity, functional and molecular proofs of this mechanism are still elusive. Herein, we have provided novel evidence that Pem after 1 dose increases circulatory Th17-type cytokines’ levels, leading to endothelial activation and microvascular coronary endothelial dysfunction, as proven by cMRI, Doppler coronary BFV mapping, and immunoblotting at 2 weeks, initiating the establishment of cardiac injury. Circulating cytokines and especially IL-17α, IL-2, and TGF-β have high predictive value on immune-related toxicities in melanoma patients receiving anti-PD-1 therapies [50]. Therefore, a causal correlation of Th17-type cytokines’ acute release, in the first week, can be associated with endothelial homeostasis disruption and can later trigger Pem-induced CVAEs. Moreover, we have shown that endothelial dysfunction, which stands as a predecessor of severe cardiac systolic dysfunction, is dose- and time-dependently aggravated by Pem’s administration and consequently leads to exacerbated coronary endothelial dysregulation and inflammation at 5 weeks. The establishment of cardiotoxicity at 5 weeks was confirmed by the elevated cTnI levels in the circulation, which was significantly increased compared to the controls and baseline at this time point. The elevation of cTnI upon the establishment of cardiotoxicity is in line with the clinical observations [48, 81]. Importantly, ICAM-1 was identified as a novel biomarker of early endothelial activation in our in vivo model.

It is generally appreciated that various manifestations of HF, including ischemic cardiomyopathy, dilated cardiomyopathy, coronary microembolization drug-related cardiotoxicity and tachyarrhythmias, share microvascular endothelial dysfunction as a common confounder [27, 29, 40]. The identification and early intervention against acute endothelial dysfunction are regarded as a pivotal modality in maintaining cardiovascular homeostasis, as endothelial cells’ resilience to acute stress factors is crucial for preventing chronic cardiac dysfunction [77]. Moreover, documentation of molecular pathways, involved in endothelial dysfunction, can enable the identification of novel druggable targets against endothelial-related functional deficits in cardiovascular diseases [39, 76]. Identification and management of Pem-related early coronary endothelial dysregulation are of great clinical value, as in a recent clinical study on ICI-induced CVAEs, vascular-driven CVAEs, such as vasovagal syncope, acute myocardial infarction and microvascular dysfunction, were observed within the spectrum of ICI-induced cardiovascular complications [3]. Therefore, the pharmacological management of ICI-derived early coronary endothelial dysregulation might serve as a druggable target for the mitigation of both cardiac dysfunction and vascular-driven CVAEs by anti-PD-1 therapy. Importantly, microvascular endothelial dysfunction is an independent predictor of cancer development and progression, as it is shown that patients with nonobstructive coronary artery diseases have a higher incidence of malignancies [34, 65]. Targeting microvascular coronary endothelial dysfunction in anti-PD-1 therapy might facilitate the parallel treatment of both cancer progression and CVAEs.

Therefore, subsequently, we challenged the prophylactic potential of high-intensity statins against Pem-induced cardiotoxicity. Our rationale for selecting statins was supported by a recent clinical study, exhibiting that statins are associated with improved antitumor efficacy of anti-PD-1 therapy in malignant pleural mesothelioma and advanced nonsmall-cell lung cancer patients [7]. We employed two statins with proven endothelial protective potential [83] and different intensities as potential cardioprotective candidates. Our in vitro human-based studies deduced that only Atorv, at a high dose, mitigated Pem-induced endothelial toxicity, while it also prevented early and late cardiac histological, functional, and molecular deficits induced by Pem in vivo. The lack of Prav’s cardioprotective potential can be accredited to the lower lipophilicity and intensity compared to Atorv leading to differential potency on the endothelium [83]. Though the putative antitumor potential of statins is already studied and might be attributed to their direct effect ontumor cells, downregulating PD-L1 [45] and suppressing tumor escape by inhibiting PD-L1 trafficking [10], statins’ effect on anti-PD-1-related CVAEs is not yet investigated. This is of great interest, considering the proven prophylactic effect, especially of Atorv, against anthracycline-induced cardiotoxicity [63]. The potential prophylactic effect of Atorv against doxorubicin-induced cardiotoxicity is previously revealed by the STOP-CA clinical trial on 300 patients with lymphoma. In this study, Atorv 40 mg reduced the incidence of cardiac dysfunction, as evaluated by left ventricular EF % decline [59]. The latter clinical trial reinforces the high prophylactic potential of Atorv in the cardio-oncology setting, regarding the anthracycline-induced cardiotoxicity. However, its impact on the cardiovascular function of ICI-treated patients is not yet investigated. Taking under consideration that anthracycline- and ICI-induced cardiotoxicity present differences in the mechanism and manifestation of CVAEs [42], our study is merited with novel findings on the prophylactic potential of Atorv also in ICI-induced cardiotoxicity, besides its already supported beneficial effect against anthracycline-induced cardiomyopathy.

Approximately 30% of patients receive statins at the start of their cancer therapy [7, 15]. In a recent study of 14,902 patients with breast cancer, it was presented that, compared with nonusers, patients receiving statins had a significantly lower risk of cancer-related mortality. The ratios of patients who experienced CVAEs, including cardiovascular death, HF, and arterial or venous events, were similar between statin users and nonusers [8]. However, it should be noted that patients receiving statins in the aforementioned study were relatively older and had a higher incidence of coronary artery disease, hypertension, and diabetes. Additionally, in the statin-receiving patients, a higher co-medication frequency with angiotensin-converting enzyme inhibitors/angiotensin receptor blockers and antiplatelet agents was observed. Therefore, a linear conclusion on the protection of cancer patients by statin therapy cannot be drawn per se and the presence of cardiovascular comorbidities and comedications seems to complicate their cardiovascular benefits. Data on the effect of different statins on CVAEs in cancer patients are scarce. Among the studies investigating the effect of statins on cancer progression, lipophilic/high-intensity statins seem to have a favorable effect [4, 46, 47]. Specifically for anti-PD-1 therapy, it is shown that only high-intensity statins improve its clinical potential in the clinical setting [7]. However, regarding their prophylactic value in patients manifesting anticancer therapy-related cardiovascular complications, both hydrophilic and lipophilic statins may also be cardioprotective during cancer therapy [26]. Since cardioprotection in cardio-oncology has raised a critical concern on the selection of cardioprotective modalities in the presence of malignancies, it can be assumed that lipophilic/high-intensity statins, such as Atorv, exhibiting concurrent cardioprotective and anticancer potential should be preferred in the cardio-oncology setting.

The clinical arsenal lacks specific prophylaxis against anti-PD-1-related CVAEs. Preclinical studies have already proposed various prophylactic therapies against the observed cardiotoxicity, extending from anti-IL-17α and anti-CD4 or anti-CD8 to anti-TNF-α therapies [22, 54]. However, the interference of the prophylaxes with the antitumor effect of anti-PD-1 therapy seems to limit the safety and efficacy of these interventions. For instance, anti-CD8 therapy abrogates anti-PD-1 antitumor potential in vivo [54]. Therefore, targeting T-cell populations to combat ICI-related cardiotoxicity appears to increase the risk of cancer relapse. Although anti-TNF-α therapy seems to mitigate the anti-PD-1-related AEs, without interfering with its antitumor effect [54], and PD-1 + TNF-α dual blockade might additionally reduce tumor resistance in vivo [5], combination therapy should be considered with caution. In the heart, it seems that TNF-α contributes to ischemia/reperfusion injury, post-myocardial infarction remodeling, and heart failure development making it a favorable target for cardioprotection [40]. Despite the fact that acute TNF-α blockade might present a short-term beneficial effect on anti-PD-1-related CVAEs, long-term TNF-α inhibition is implicated with CD8 + -T-cell senescence and toxicity which might lead to a possible cancer relapse [9]. Therefore, extensive short- and long-term clinical trials must be carefully designed and implemented for the establishment of a solid beneficial potential of the aforementioned combination. Additionally, the pharmacokinetics and pharmacodynamics of the drugs in the combination regimens should be scrutinized in future clinical studies. Herein, we provide for the first time solid evidence that Atorv, a widely used drug that does not interfere with the antitumor effect of Pem (as it has been shown in clinical studies) [7], can prevent anti-PD-1-related cardiotoxicity.

In our study, male mice were used for the conduction experiments. The use of male animals was selected, as male mice do not present the hormonal fluctuations due to the menstrual cycle observed in female mice, leading to difficulties in data interpretation and increased variability of the results. However, according to sex relevance in cardio-oncology studies [1], this is a limitation of the study and future studies should be conducted to investigate the sex differences in Pem-induced cardiotoxicity. Another shortcoming of the current study is that data were not validated in a tumor-bearing in vivo model. There is an imperative need to understand the pivotal biological crosstalk between cardiovascular morbidities and malignancies, as on the one hand they may enable the development of novel therapeutic and preventive modalities for both diseases [52], whereas on the other hand they may reveal novel challenges for cardioprotection [28]. Despite the fact that prophylactic therapies against new-onset HF in cancer patients have been extensively investigated, studies and indications on cancer management in patients with preexisting HF and data on whether guideline-directed medical therapy for HF should be modified upon cancer diagnosis are still obscure and need further investigation [73]. It is described that endothelial dysfunction and cancer might share common confounders, namely activation of the Wnt signaling pathway and depression of peroxisome proliferator-activated receptor gamma (PPAR gamma) signaling [53], which might link endothelial microvascular dysfunction and cancer. These links can facilitate the identification of high-risk individuals for developing malignancies and may permit the improved insight from healthcare providers to risk-stratify these patients. Also, they might further support the concept of joint pharmacologic strategies against cardiovascular diseases and cancer [52]. Besides the interplay of cancer and microvascular endothelial dysfunction, it is well-known that cancer has a direct negative impact on the myocardium. Cancer itself may pose a major burden to cardiovascular homeostasis, with a significant impact on the manifestation of CVAEs, whereas cardiovascular disease may also accelerate tumor progression [55]. The interplay between cancer and cardiovascular outcomes is also evident in the clinical arena, as lung cancer patients with high tumor burden, receiving ICI therapy, manifested more frequently severe immune-related adverse events, than the low tumor burden individuals [71]. Therefore, the validation of our data on a murine model of malignancy is of utmost importance, regarding the complex regulatory circuits between the tumor, endothelial cells and cardiac dysfunction, which will be investigated in future preclinical and clinical studies. Finally, to the best of our knowledge, cardio-oncology preclinical and clinical studies have not yet identified the optimal dose regimen for atorvastatin cardioprotection, while only the high doses of statins are investigated in contemporary preclinical and clinical studies [20, 59, 61]. Additional studies on statins’ dose titration, for achieving prophylaxis against anticancer agent-related cardiotoxicity, should be performed. In our study, only the high translational dose of atorvastatin was investigated according to previous preclinical studies [20, 61]. However, the lack of atorvastatin dose titration in our in vivo model of Pem-induced cardiotoxicity is a limitation of the study.

## Conclusions

**Fig. 10:**
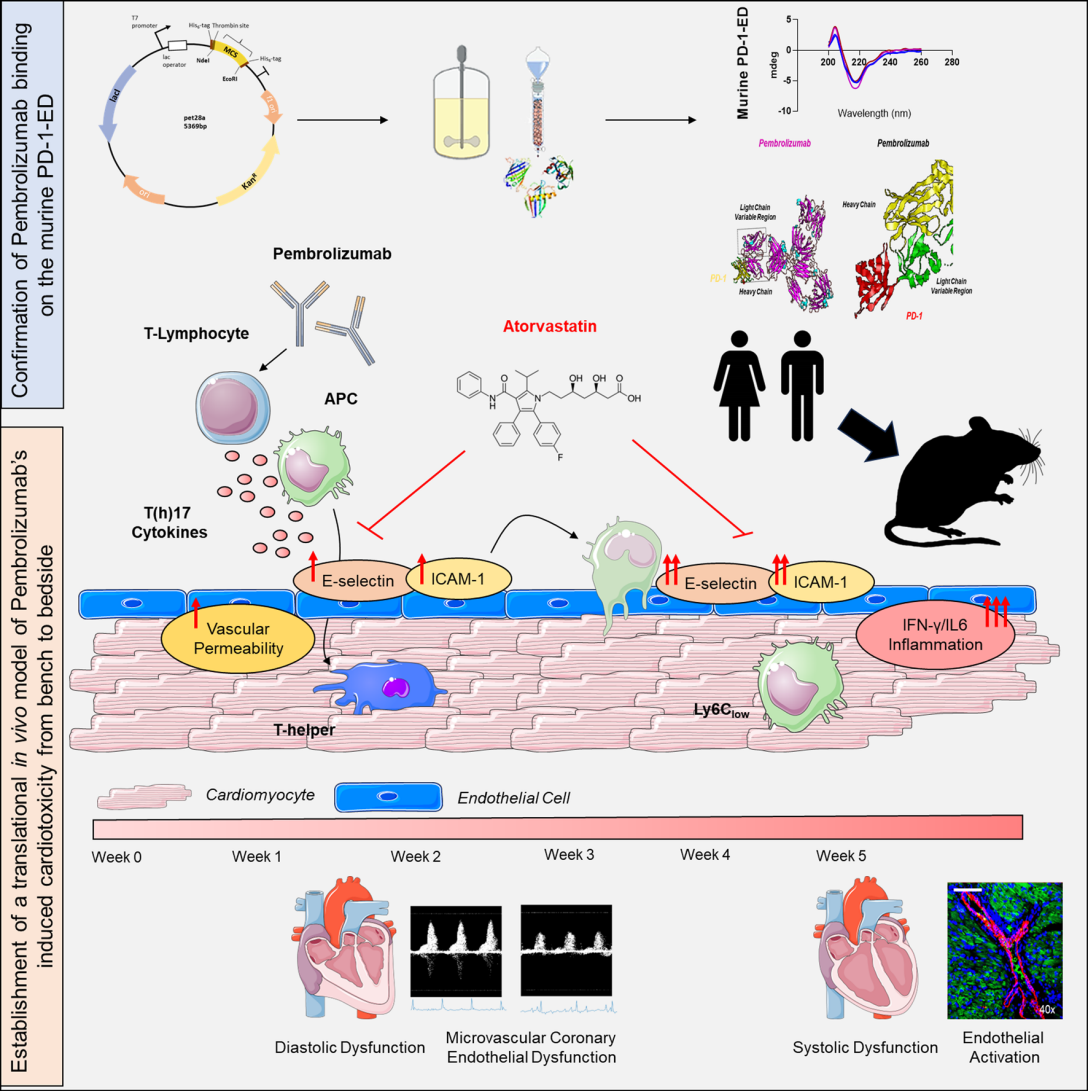
Bedside-to-bench investigation of pembrolizumab-induced cardiotoxicity. Prophylactic potential of atorvastatin. Central illustration of the key findings of the study depicting the confirmation of pembrolizumab’s binding to the murine PD-1-ED, the establishment of the in vivo model of pembrolizumab-induced cardiotoxicity, and the identification of the prophylactic role of atorvastatin. *ICAM-1* intracellular adhesion molecule 1*, IFN-γ* interferon gamma*, IL-6* interleukin 6*, PD-1-ED* programmed death 1 extracellular domain. Images were generated using templates from Servier Medical Art, licensed under a Creative Commons Attribution 3.0 Unported License

Our findings provide novel in vitro and in vivo evidence of Pem’s cross-reactivity with the murine PD-1-ED, supporting the conduction of new translational studies on anti-PD-1 therapies. Pem was found on a histological, functional, and molecular level to induce immune-related early endothelial activation and microvascular coronary endothelial dysfunction, whereas ICAM-1 emerged as a novel biomarker of Pem’s cardiotoxicity (Fig. 10). Atorv emerges as a novel cardioprotective modality, which successfully abrogated early and late signs of Pem-induced cardiotoxicity. Further clinical studies are required for the establishment of the dual anti-PD-1 and Atorv therapy as a new cornerstone in cancer immunotherapy.

Cardio-oncology prophylactic and therapeutic interventions are limited and primarily focus on the management of anthracyclines-induced cardiotoxicity. It is already shown that multiple cardioprotective agents, including Atorv and cardioprotective maneuvers, such as remote ischemic preconditioning can mitigate anthracycline-induced cardiotoxicity in the preclinical and clinical setting [31]. Moreover, the application of cardioprotective strategies in the cardio-oncology setting should be considered with caution, regarding the effect of the cardioprotection on tumor progression. Therefore, translational preclinical models and carefully designed trials are of imperative need [28].

## Supplementary Information

Below is the link to the electronic supplementary material.

Supplementary file1 (DOCX 1438 KB)

## Data Availability

The authors declare that the data supporting the findings of this study are available within the article and in the supplemental information files. Additionally, data will be provided upon request.

## Abbreviations

Ach: Acetylcholine
ANOVA: Analysis of variance
ARRIVE: Animal research reporting of in vivo experiments
Atorv: Atorvastatin
Ave: Avelumab
BFV: Blood flow velocity
CD: Circular dichroism
CD4: Cluster differentiation 4
CD44: Cluster differentiation 44
CD8: Cluster differentiation 8
CK-MB: Creatine phosphokinase-MB
cMRI: Cardiac magnetic resonance imaging
cTnI: Cardiac troponin I
CTLA-4: Cytotoxic T lymphocyte-associated antigen 4
CVAEs: Cardiovascular adverse events
*E. coli*: *Escherichia coli*
E/A ratio: Left ventricular relaxation in early diastole (E wave) to peak velocity flow in late diastole in atrial contraction (A wave) ratio
ED: Extracellular domain
EF %: Ejection fraction %
EMA: European medicines agency
eNOS: Endothelial NO synthase
FDA: Food and Drug Administration
HF: Heart failure
IC: Immune cell
IC_50_: Half maximal inhibitory concentration
ICAM-1: Intercellular adhesion molecule 1
ICIs: Immune checkpoint inhibitors
IFN-γ: Interferon-γ
IL-10: Interleukin 10
IL-17α: Interleukin 17α
IL-2: Interleukin 2
IL-4: Interleukin 4
IL-6: Interleukin 6
iNOS: Inducible NO synthase
Ipi: Ipilimumab
LAG-3: Lymphocyte activation gene 3
LDH: Lactate dehydrogenase
NO: Nitric oxide
Padi4: Protein arginine deiminase type 4
pAVCs: Primary adult ventricular cardiomyocytes
PBMCs: Peripheral blood mononuclear cells
PD-1: Programmed death 1
PD-L1: Programmed death ligand 1
Pem: Pembrolizumab
PPAR gamma: Peroxisome proliferator-activated receptor gamma
STAT3: Signal transducer and activator of transcription 3
TGF-β: Transforming growth factor beta
Th17: T helper 17 cell
TNF-α: Tumor necrosis factor alpha
VCAM-1: Vascular cell adhesion molecule 1
