# Supplementary material for: Early microvascular coronary endothelial dysfunction precedes pembrolizumab-induced cardiotoxicity. Preventive role of high dose of atorvastatin
Source: Basic Res Cardiol. 2024 Mar 23;120(1):263–86. doi: 10.1007/s00395-024-01046-0 (PMC11790778; doi:10.1007/s00395-024-01046-0)
Supplement: Supplementary file 1 — Supplementary file1 (DOCX 1438 KB) [file 395_2024_1046_MOESM1_ESM.docx]

**Early microvascular coronary endothelial dysfunction precedes Pembrolizumab-induced cardiotoxicity. Preventive role of high dose of Atorvastatin.**

Panagiotis Efentakis^1^, Angeliki Choustoulaki^1†^, Grzegorz Kwiatkowski^2†^, Aimilia Varela^3^, Ioannis V. Kostopoulos^4^, George Tsekenis^3^, Ioannis Ntanasis-Stathopoulos^5^, Anastasios Georgoulis^1^, Constantinos E. Vorgias^6^, Harikleia Gakiopoulou^7^, Alexandros Briasoulis^5^, Constantinos H. Davos^3^, Nikolaos Kostomitsopoulos^3^, Ourania Tsitsilonis^4^, Meletios Athanasios Dimopoulos^5^, Evangelos Terpos^5^, Stefan Chłopicki^2,8‡^, Maria Gavriatopoulou^5‡^, Ioanna Andreadou^1‡#^

1. Laboratory of Pharmacology, Faculty of Pharmacy, National and Kapodistrian University of Athens, Athens, Greece.
2. Jagiellonian Centre for Experimental Therapeutics (JCET), Jagiellonian University, Kraków, Poland.
3. Biomedical Research Foundation of the Academy of Athens, Athens, Greece.
4. Flow Cytometry Unit, Section of Animal and Human Physiology, Department of Biology, National and Kapodistrian University of Athens, Athens, Greece.
5. Department of Clinical Therapeutics, School of Medicine, National and Kapodistrian University of Athens, Athens, Greece.
6. Department of Biochemistry & Molecular Biology, Faculty of Biology, National and Kapodistrian University of Athens, Athens, Greece.
7. 1st Department of Pathology, School of Medicine, National and Kapodistrian University of Athens, Athens, Greece.
8. Chair of Pharmacology, Jagiellonian University, Medical College, Krakow, Poland

*†, Equally contributed second authors ‡ Equally contributed senior authors*

*#* ***Corresponding author****: Ioanna Andreadou, Faculty of Pharmacy, 1. Laboratory of Pharmacology, Panepistimiopolis, Zografou, Athens 15771, Greece; tel: +30 210 7274827; fax: +30 210 7274747; e-mail:* [*jandread@pharm.uoa.gr*](mailto:jandread@pharm.uoa.gr)

**Supplemental Methods**

**Murine primary adult ventricular cardiomyocytes isolation and *in vitro* experiments**

Murine primary adult ventricular cardiomyocytes (pAVCs) were isolated as described [6] from C57Bl/6 mice (12–14 weeks of age; n = 6). pAVCs were cultured for 18h in culture medium conditioned with ICIs, namely Pembrolizumab (Pem), Ipilimumab (Ipi) and Avelumab (Ave) (0-100μg/ml). The incubation time of 18h was chosen as the least compromising time point of viability of untreated pAVCs and the most pronounced effect of the treatments [6]. The concentration of the ICIs was selected according to the pharmacokinetic data of the ICIs in humans [7, 10, 11]. Subsequently, cell viability was assessed directly through 3-(4,5-dimethylthiazol-2-yl)-2,5-diphenyl-2H-tetrazolium bromide (MTT) assay.

**Primary Splenocytes Isolation**

Primary splenocytes, consisting mainly from immature B- and T-cells [2], were isolated from C57Bl/6 mice (12–14 weeks of age; n = 5-6) via mechanical disruption of the murine spleen through a 40μm cell strainer mesh (#93040, SPL Life Sciences, Korea) as previously described [21]. Primary splenocytes were treated with ICIs at a concentration range of 0-100μg/ml and their viability was assessed by MTT assay after 24h. Moreover, their ICI-conditioned media were transferred onto murine pAVCs for 18h for the assessment of the immune cell (IC)-mediated toxicity by the ICIs.

**Real-Time Polymerase Chain Reaction**

For RNA isolation, pAVCs and splenocytes were washed once with PBS after the respective treatments, collected by scraping and extracted by the standardized Trizol protocol. RT-PCR was performed with the CFX96 Real-Time PCR detection system (Bio-Rad, Munich, Germany). Isolated RNA was reverse-transcribed to cDNA using high-capacity cDNA reverse transcription kit (FastGene Scriptase II cDNA, Nippon Genetics, Japan). Specific primer pairs (Eurofins Genomics AT, GmbH) were designed (Primer-Blast, NCBI, NIH) and used in order to detect mRNA expression of target genes, using the SYBR® Green method (Eva Green, Solis BioDyne, Estonia) according to the manufacturer’s instructions [5]. Real-time PCR primers’ sequences are presented in **Supplemental Table 6**.

**Peripheral Blood Mononuclear Cell isolation**

Peripheral blood mononuclear cells (PBMCs) were isolated using Lymphosep (density 1.077 g/ml, # LM-T1702/100, Biosera, Cholet, France) from freshly collected blood from healthy volunteers; blood was first diluted (1:1) with phosphate-buffered saline (PBS) and was carefully layered over the separation medium (half the volume of the sample) and the two phases were kept separated before centrifugation. Samples were centrifuged at 400 x g for 30min at 20°C. PBMCs were collected after aspiration of the plasma and platelets layer; cells were then washed with PBS and processed for downstream assays [4].

**Human Peripheral Blood Mononuclear Cell and EAHy-926 cell culture**

Human PBMCs were cultured in Roswell Park Memorial Institute (RPMI-1640) medium, supplemented with 1% penicillin/streptomycin (P/S) in the presence of Pem (100μg/ml) for 72h, according to prior *in vitro* reports [25]. The activation of PBMCs was confirmed by immunofluorescence microscopy using Cluster Differentiation Molecule 44 (CD44), inducible nitric oxide synthase (iNOS) and Interferon gamma (IFN-γ) as activation markers [1]. Pem-conditioned media from PBMCs were transferred onto EAHy-926 human endothelial cells and their viability was assessed directly by MTT assay after 24h. EAHy-926 cells are a somatic hybrid cell derived from human endothelium and A549/8 cells. They display stable endothelial characteristics and may be used in cardiovascular research [28]. Cells were grown in Dulbecco's Modified Eagle Medium (DMEM, high glucose, 1%P/S, 10% Fetal Bovine Serum) until confluency. Endothelial cells were starved 24h prior to medium transfer and were seeded in 96- or 6-well plates for viability or molecular assays respectively. The half maximal inhibitory concentration (IC_50_) of cellular viability for Atorvastatin (Atorv) and Pravastatin (Prav) was performed at 0.5, 5, 50, 500 and 5000 μΜ (concentration range 0.5μΜ-5mM), whereas the final tested concentrations (5-10μΜ) were selected according to literature [3].

**Recombinant expression and purification of the human and murine PD-1 Extracellular Domain**

Total RNA from one healthy volunteer’s PBMCs and primary murine splenocytes were isolated. The cDNA fragments encoding the extracellular domain (ED) of human and murine PD-1 (aa 25-167) were amplified by PCR (CFX Connect Real-Time PCR Detection System, Bio-Rad Laboratories, California, USA) using suitably designed primers (**Supplemental** **Table 7**) containing EcoRI and NdeI restriction sites (**Fig. 1a**). The resulting PCR products were cloned into the pet28a vector (**Fig. 1b**) upon digestion with EcoRI (#1040A, Takara, Japan) and NdeI (#1161A, Takara, Japan) restriction enzymes and ligation with DNA ligase (#2011A, Takara, Japan), as previously described [16, 19]. *E. coli* expression cells (BL21-GOLD(DE3)) were transformed with the recombinant vectors [16]. A fresh colony was chosen to grow in a 5mL starter culture in Luria Broth (LB) medium (#L3022, Sigma Aldrich/Merck, Darmstadt, Germany) overnight at 37°C and 180 rpm. All media were supplemented with 50μg/mL kanamycin (#15321, Cayman Chemical, Michigan, USA). The next day, the starter culture was transferred into 1L of fresh LB medium, until cells reach an OD_600_ of 0.6. For induction, isopropyl-beta-d-thiogalactopyranoside (IPTG) (#BIMB1008, Apollo Scientific, UK) was added to a final concentration of 1mM and the culture was grown for 3h at 37°C and 180 rpm (**Fig. 1c, d**) [15, 16]. Cells were subsequently harvested by centrifugation (6.000 x g, 15mins, 4°C). The cell pellet was resuspended in ice-cold lysis buffer (20mM Tris, 200mM NaCl, 10mM imidazole, 8M urea, pH=8) and lysed by sonication on ice in the presence of 1mM protease inhibitor phenylmethylsulfonyl fluoride (PMSF) (#52332, Sigma Aldrich/Merck, Darmstadt, Germany). Both proteins were expressed as inclusion bodies demanding the use of 8M urea for further solubilization [15, 27]. The insoluble material was pelleted by centrifugation (15000 x g, 30min, 4°C) and removed. The protein solution, containing the target protein as a His-tagged protein, was applied to Ni^+2^-NTA column (1mL bed volume) (#745400, Protino® Ni-NTA Agarose, Macherey-Nagel, Germany) previously equilibrated with the same buffer. The column was washed with fifty bed volumes of wash buffer 1 (20mM Tris, 200mM NaCl, 20mM imidazole, 8M urea, pH=8) and ten bed volumes of wash buffer 2 (20mM Tris, 200mM NaCl, 50mM imidazole, 8M urea, pH=8), as instructed by the manufacturer. The protein was then eluted with elution buffer (20mM Tris, 200mM NaCl, 250mM imidazole, 8M urea, pH=8). The eluted protein was subsequently loaded onto a Ni^+2^-NTA column to achieve on-column refolding and further purification using a linear gradient of 8M-0M urea and 10mM-250mM imidazole [26, 30]. The quantity and purity of the protein in each elution was evaluated by SDS-PAGE electrophoresis (**Fig. 1e, f**). The elusions with the highest purity of protein were selected and subjected to buffer exchange by dialysis against 20mM Tris, 200mM NaCl, pH=8 [15], a buffer suitable for the following Circular Dichroism (CD) experiments [8].

**Circular Dichroism Spectroscopy**

Far-UV circular dichroism (CD) measurements were conducted using a CD spectrophotometer (J-1500, JASCO, Easton, USA) connected to a Peltier temperature controller. An average of three scans was obtained, using a quartz cuvette with a 1mm or 2mm path length, for structural and binding studies respectively. Data was collected every 0.5nm with an averaging time of 1s. The spectral bandwidth was 1nm. To assess structural stability of the recombinant proteins, measurements were conducted at 15, 25 and 37^o^C. To investigate structural rearrangement of Pem upon binding, recombinant human and murine PD-1-ED was added to an equal concentration to the antibody solution (1:1) at 0.6μΜ and CD measurements were conducted at 15^o^C and 37^o^C. The acquired spectra were analyzed using BeStSel software to determine secondary structure content [17].

***In silico* protein-protein docking experiments**

*In silico* murine PD-1-ED and Pem binding experiments were conducted with ClusPro 2.0 protein-protein docking online software [13]. Crystal structure of the murine PD-1-ED (1NPU, RCSB, Protein Data Bank) [29] and crystal structure of Pem (5DK3, 1NPU, RCSB, Protein Data Bank) [20] were used to generate murine PD-1-ED and Pem binding models. Cluster-0 and -1 models with lowest binding energy were chosen following the already published data of Pem binding on the human PD-1-ED [18].

**Echocardiography**

Echocardiographic analysis was performed in anesthetized mice with isoflurane (5% in 1L/min oxygen for induction, and 1% for maintenance of anesthesia). Transthoracic echocardiography was performed by an experienced sonographer in a blinded manner with a high-frequency ultrasound imaging system (Vevo 2100; Visualsonics Inc., Toronto, ON, Canada) equipped with 18-38MHz linear-array transducer (MS400). Heart rate, left ventricular end-diastole (LVEDD) and left ventricular end-systole diameter (LVESD), left ventricular posterior wall thickness at diastole (LVPWd) and at systole (LVPWs), fractional shortening (FS; FS% = (LVEDD - LVESD)/LVEDD×100%) and ejection fraction % (EF; EF%= ((LVEDD^3^−LVESD^3^)/LVEDD^3^×100%) and left ventricular radius to left ventricular posterior wall thickness ratio were calculated [4].

**Circulating biomarkers of cardiac damage assessment**

Circulating biomarkers of cardiac damage were assessed in plasma samples of the IgG4- and Pem-treated mice. Cardiac Troponin I (cTnI), lactate dehydrogenase (LDH) and creatinine phosphokinase-MB (CK-MB) were measured according the manufacturers’ instructions (cTnI: #MOEB0394, Assay Genie, Ireland; LDH: #001698; CK-MB; 001663, Biosis, Greece). cTnI is presented as pg/mL, whereas LDH and CK-MB as U/L.

**Magnetic Resonance Imaging**

All magnetic resonance (MR) experiments were recorded with a 9.4T small animal MRI scanner (Bruker BioSpec, Ettlingen, Germany) equipped with a 1000mT/m gradient coil with a maximum slew rate of 3500 T/m/s. A 36mm quadrature volume coil was used for RF excitation and detection. For imaging, the animals were placed in a prone position, with the heart in the center of the detection coil. Anesthesia was delivered via a nose cone at a constant level of 1.75% isoflurane in a mixture of 0.4l/min O_2_: 0.8 l/min air. Body temperature was monitored with an endorectal probe and maintained in the range of 35.5°C–36.5°C. To evaluate left ventricle chamber volumes, the bright-blood cine images were collated in 7–9 contiguous slices covering the whole ventricle volume using a flow-compensated, prospectively gated gradient-echo FLASH sequence with the following parameters: FOV 30 x 30mm^2^, acquisition matrix: 128 x 128, TE/TR = 2.3/5ms, slice thickness=1 mm, number of averages = 4, flip angle = 11 ͦ. Depending on the heart rate, between 22 and 24 cine frames were acquired. The LV function was acquired with high-temporal resolution, retrospectively gated cine FLASH GRE sequence (IgFLASH) in a mid-ventricular, a short-axis mid-ventricle and a long-axis, four-chamber slice. The following acquisition parameters were used: FOV 30 x 30mm^2^, acquisition matrix 128 x 128, TE/TR = 1.3/4.2ms, slice thickness=1mm, number of repetitions = 1600, flip angle = 11 ͦ. Data were reconstructed to 60 frames per cardiac cycle using a vendor-provided macro (ParaVision 6.0.1, Bruker BioSpin, Ettlingen, Germany). End-systolic (ESV) and end-diastolic (EDV) volumes, stroke volume (SV), ejection fraction (EF), and cardiac output (CO) were obtained by summing LV volume from adjacent short-axis slices covering the whole LV. A time-volume curve (TVC) was calculated from LV volumes (including papillary muscles) assessed using short-axis semiautomatic segmentation with high-temporal resolution data sets. A piecewise linear regression (PLR) implemented in MATLAB (MathWorks), as described previously [24], was used to obtain ejection (ER) and filling rates (FR), with slopes of segments fitted by TVC normalized to the individual SV and RR intervals. Duration of ejection (ET), isovolumic relaxation (IVRT), filling (FT) and isovolumic contraction phases (IVCT) were taken from the PLR model and normalized to the RR interval. The early (E-peak) and atrial (A-peak) were calculated by taking a first derivative of the TVC curve [22]. Radial and circumferential peak strain were calculated using a short-axis mid-ventricular slice, whereas longitudinal peak strain was calculated using a four-chamber, long-axis slice. The strain calculations were performed using Segment (Medviso, Lund, Sweden) version 3.2 R8351 (<http://segment.heiberg.se>) [9, 14].

**Coronary artery flow reserve assessment**

Coronary artery blood flow velocity mapping was performed during rest and maximal vasodilation in response to adenosine and acetylcholine infusion. A doppler flow velocity system (Indus Instruments, Texas, USA) equipped with a single transceiver 20-MHz Doppler probe was used. Anesthesia was delivered via a nose cone at a level of 1.25%–1.0% isoflurane in a mixture of 40% O_2_:80% air. The animals were placed in a supine position on a heating pad and secured to a four-channel ECG system with tape on each paw. An endorectal probe was inserted for continuous body temperature monitoring. A cannula was placed into a tail vein for injection vasodilators. The upper chest was shaved with an electric razor and ultrasound gel was applied to both the chest and the Doppler probe. The probe was manually adjusted and secured with a micromanipulator. The correct position of the probe along the coronary artery was confirmed with a visual inspection of the blood flow velocity waveform and its relation to the ECG signal [14]. The basal blood flow velocity was recorded after approximately 10min of animal rest after anesthesia induction. Next, the hyperemic response was measured after 2min of continuous infusion with adenosine at the dose of 144μg/kg^/^min, 10μL/min (Sigma Aldrich Chemie GmbH, Steinheim, Germany). Subsequently, 15min after the adenosine infusion stop, hyperemic response after 2min of continuous infusion of acetylcholine at the dose of 2μg/kg^/^min, 10μL/min (Sigma Aldrich Chemie GmbH, Steinheim, Germany) was measured. The intravenous cannula was flushed with saline in between the infusions to prevent any contamination from residual adenosine in the infusion line. At each time point, blood flow velocity was recorded for 2000ms, comprising 16–20 blood flow speckles. Speckles recorded during expiration were included in the analysis, and the average overall speckles were taken at the maximum value of blood flow velocity. Coronary flow reserve was calculated as the ratio between blood flow velocity after adenosine/acetylcholine infusion to a pre-injection, rest value.

**Histology**

Myocardial tissue samples for histopathological evaluation were fixed in 4% buffered formalin solution for 24h and embedded in paraffin waxes. Three-micrometer-thick sections were obtained from each sample and routinely counterstained with hematoxylin and eosin for histological examination. The latter was performed under light microscope (Nikon Eclipse 80i, Nikon Corp, Tokyo) individually by two pathologists taking into account nuclear shape irregularity, vessel congestion and edema, myocytolysis and possible interstitial fibrosis.

**Flow cytometry**

The flow cytometric analysis of the whole blood and heart was performed as previously described [23]. Whole blood was obtained by cardiac puncture and collected in tubes containing ACD (citrate-dextrose solution) as anticoagulant. The red blood cells were lysed with NH_4_Cl solution for 5 min at room temperature under agitation. Cell suspension was centrifuged at 300 x g for 5 min at 4°C and the pellet was washed with cold PBS supplemented with 0.5% (w/v) bovine serum albumin (BSA). Myocardium was excised and digested with a mixture of collagenase type I (1mg/mL; #SCR103, Sigma-Aldrich, Darmstad, Germany), DNase I (50U/mL; #E091, Applied Biological Materials, Canada) and hyaluronidase (300 μg/mL; #HX0514, Sigma-Aldrich), in PBS containing 20 mM HEPES for 60 min at 37°C. The cell suspension obtained was filtered through a 70μm strainer and lysed with a NH_4_Cl red blood cell lysing solution for 3 min. Cells were centrifuged at 400 x g for 10 min, resuspended with cold PBS containing 2% fetal bovine serum (FBS) and evaluated for cell density and cell viability with Trypan-Blue staining using a hemocytometer. The minimum viability cut-off for further processing was at 90%. Cells were then incubated with Fc-block anti-CD16/32 mAb (#553142, BD Pharmingen, San Diego, CA, USA) for 15 min at 4°C to prevent unspecific binding before staining with anti-Ly6G-PE (#551461, clone 1A8, 1:400, BD Pharmingen), anti-Ly6C-FITC (#553104, clone AL-21, 1:200, BD Pharmingen), anti-CD11b-APC (#553312, clone M1/70, 1:400, BD Pharmingen), anti-CD19-PE-Cy7 (#552854, clone 1D3, 1:400, BD Pharmingen), anti-CD3e-PerCP-Cy5.5 (#551163, clone 145-2C11, 1:200, BD Pharmingen), anti-CD4-BV510 (#563106, clone RM4-5, 1:1000, BD Pharmingen), anti-CD8a-APC-Cy7 (#557654, clone 53-6.7, 1:400, BD Pharmingen) for 30 min at 4°C. Stained cells were then centrifuged at 300 x g for 5 min at 4°C, washed twice with PBS-0.5% FBS and processed immediately for acquisition on a 3-laser BD FACSCanto II cytometer with a FSC threshold set at 10.000. A minimum of 10^6^ total events were acquired for each heart sample and data analysis was conducted with the BD FACSDiva software. The gating strategy is illustrated in **Supplemental Fig. 4**.

**T helper 17-type Cytokine Profile**

The Th17-type cytokine profile was assessed with flow cytometry using a commercially available bead-based multiplex assay kit, using fluorescence–encoded beads, according to the manufacturer’s instructions (#741047, LEGENDplex™ MU Th17 Panel (7-plex) w/ FP V03, Biolegend, UK). Acquisition was performed on a BD FACS Canto II cytometer and analysis was assessed with the proposed LEDENDplex software. Seven Th17-cell secreted cytokines, namely IFN-γ, TNF-α, IL-6, IL-10, IL-17a, IL-17F, IL-22 were simultaneously quantified in the sera of the IgG4- and Pem-treated mice at baseline, at 1^st^, 2^nd^ and 5^th^ week of administration (n=6) and in the Atorv cohort at 1^st^ week of administration.

**Western Blot Analysis**

Western Blot analysis in myocardial tissue and *in vitro* samples was performed as described previously [4]. Snap-frozen myocardial tissue samples or cells were lysed in lysis buffer containing 1% Triton-X, 20mM tris(hydroxymethyl)aminomethane (Tris-Base) (adjusted pH 7.4), 150mM NaCl, 50mM KF, 1 mM ethylenediaminetetraacetic acid (EDTA), 1mM ethylene glycol-bis(β-aminoethyl ether)-N,N,N',N'-tetraacetic acid (EGTA), 1% sodium dodecyl sulphate (SDS), 0.5% sodium deoxychollate and 0.1% protease/phosphatase inhibitor cocktail, and homogenized. Lysates were centrifuged for 15min (13000 x g, 4°C). Supernatants were used for the determination of total protein by the Lowry method and samples preparation with Dave’s buffer (4% SDS, 10% 2-mercaptoethanol, 20% glycerol, 0.004% bromophenyl blue, and 0.125M Tris–HCl), as previously described [4]. An equal amount of protein was loaded into each well and then separated by suitable SDS-PAGE concentration and transferred onto a polyvinylidene difluoride membrane (PVDF), which was blocked for 1-2 hours with 5% non-fat dry milk. Membranes were incubated overnight (4°C) with primary antibodies and for 2 hours with secondary horseradish peroxidase (HRP)-conjugated antibodies at room temperature. Probed membrane signals were detected using chemiluminescent HRP substrate (Luminata Forte Western HRP substrate, Millipore, USA) and imaged using an automated cooled charge-coupled device imager (ImageQuant LAS 500, GE Healthcare Bio-Sciences, Sweden). Relative densitometry was determined among groups using ImageJ 1.49v software (National Institutes of Health, USA).

Primary antibodies against phospho-eNOS (Ser1177, #9571), eNOS (#32027), iNOS (#13120), α-actinin (#3134), VCAM-1 (#39036), phospho-STAT-3 (#9145), STAT-3 (#4904), IFN-γ (#98139) and IL-6 (#12912) (Cell Signaling Technology, Europe, B.V.), ICAM-1 (# MA5407, Invitrogen, Thermo-Fischer Scientific, Waltham, MA USA), E-selectin (#ab18981, Abcam, Cambridge, UK) were used at 1:1000 dilution. Secondary HRP-linked antibodies anti-mouse and anti-rabbit were used (#7076, #7074 Cell Signaling Technology, Europe, B.V.) for protein visualization at 1:2000 dilution.

**Immunofluorescence and Confocal Microscopy**

Cells were washed once with PBS and fixed in 4% paraformaldehyde (PFA). Subsequently, cells were permeabilized with 0.1% TritonX in PBS, and non-specific binding of the antibodies was blocked by incubation for 1h in 1% BSA-0.01% Tween-80 in PBS. Myocardial cryosections (5μm) were fixed with 4% PFA, permeabilized with 0.25% TritonX in PBS and blocked with 3% BSA-0.01% Tween-80 in PBS. Subsequently, samples were incubated with ICAM-1 (# MA5407, 1:200, Invitrogen, Thermo-Fischer Scientific, Waltham, MA USA), IFN-γ (#98139, 1:200, Cell Signaling Technology, Europe, B.V.) or TnI (#13083, 1:400, Cell Signaling Technology, Europe, B.V.). Primary antibodies were incubated overnight, washed off with PBS and subsequently anti-rabbit/Alexa-Fluor 647 (Donkey, # ab150079, Abcam), anti-mouse/Alexa-Fluor 647 (Donkey, #ab150107, Abcam) conjugated secondary antibodies were added. Where applicable, phalloidin (Alexa Fluor™ 488 Phalloidin, Invitrogen, Thermo-Fischer Scientific, Waltham, MA USA) was applied for 10min according to manufacturer’s instructions. After washing twice in PBS, specimens were treated with anti-fade mount medium containing DAPI (P36962, Thermo Fisher Scientific) and visualized in a confocal laser-scanning microscope (Leica SP8 confocal microscope, 40×/63×, dry or oil immersion objective). Z stacks were obtained were generated using Fiji-Image J software 18. At least 6 different areas were imaged per slide and results were averaged into a single n value. Images to be quantified were acquired and exported in 12-bit grayscale format and aberrations were corrected using Fiji-Image J software. Relative quantitation was performed in images with even illumination across the field. Control slides with positive signal for each fluorophore were used for each staining to apply corrections. Constant acquisition settings were maintained among the samples with the same staining. The lowest laser power that provided a sufficient signal-to-noise ratio was used in every imaging. Fluorophore intensity was represented as integrated fluorescence density in the figures and was normalized to the DAPI signal of the respective image. Acquisition of the images were performed according to confocal microscopy guidelines [12].

**Supplemental Tables**

**Supplemental Table 1: Estimated secondary structure content % analysis of the CD spectrum of the extracellular domain of murine and human PD-1 acquired at 15°C using the BeStSel software to determine secondary structure content.**

| **Supplemental Table 1** | | | |
| --- | --- | --- | --- |
| **Extracellular domain of murine PD-1** | | | |
| Estimated secondary structure content % | | | |
| **Helix** | 8.6 | **Helix1 (regular)** | 3.2 |
|  |  | **Helix2 (distorted)** | 5.4 |
| **Antiparallel** | 28.9 | **Anti1 (left-twisted)** | 1.1 |
|  |  | **Anti2 (relaxed)** | 11.9 |
|  |  | **Anti3 (right-twisted)** | 15.9 |
| **Parallel** | 2.2 |  | |
| **Turn** | 13.8 |  |  |
| **Others** | 46.5 |  |  |
| **RMSD** 0.0565 | | **NRMSD** 0.03629 | |
| **Extracellular Domain of human PD-1** | | | |
| Estimated secondary structure content % | | | |
| **Helix** | 5.3 | **Helix1 (regular)** | 2.2 |
|  |  | **Helix2 (distorted)** | 3.1 |
| **Antiparallel** | 32.0 | **Anti1 (left-twisted)** | 1.8 |
|  |  | **Anti2 (relaxed)** | 13.8 |
|  |  | **Anti3 (right-twisted)** | 16.5 |
| **Parallel** | 0.0 |  | |
| **Turn** | 15.0 |  |  |
| **Others** | 47.7 |  |  |
| **RMSD** 0.0426 | | **NRMSD** 0.02534 | |

*CD spectral analysis of the extracellular domain of murine and human PD-1*

**Supplemental Table 2: Echocardiography analysis at baseline in the Control and Pem groups.**

| **Supplemental Table 2** | **Control (IgG4)** | **Pem** | **p values** |
| --- | --- | --- | --- |
|  | **n=9** | **n=9** |  |
| **HR** | 567.77±37.44 | 546.88±40.23 | 0.2710 |
| **LVEDD (mm)** | 3.22±0.27 | 3.44±0.15 | 0.0670 |
| **LVESD (mm)** | 1.74±0.15 | 1.87±0.12 | 0.0587 |
| **PWTd (mm)** | 0.81±0.03 | 0.79±0.03 | 0.0628 |
| **PWTs (mm)** | 1.30±0.03 | 1.29±0.03 | 0.4626 |
| **FS%** | 45.90±1.53 | 45.50±1.65 | 0.6070 |
| **EF%** | 91.8±1.83 | 91±1.8 | 0.6231 |
| **r/h** | 2.04±0.24 | 2.19±0.09 | 0.0947 |

*Echocardiography Analysis at baseline in the Pembrolizumab cohort. Values are presented as mean ± SD. HR: Heart Rate; LVEDD: Left Ventricular End Diastolic Diameter (mm); LVESD: Left Ventricular End Systolic Diameter (mm); PWTd: Posterior Wall Thickness diastole (mm);* *FS%: % Fractional Shortening, EF%: % Ejection Fraction; r/h: ratio of LV radius to PWT-posterior wall thickness. Student’s T-Test, unpaired, two-way.*

**Supplemental Table 3: Echocardiography analysis at 5 weeks in the Control and Pem groups.**

| **Supplemental Table 3** | **Control (IgG4)** | **Pem** | **p values** |
| --- | --- | --- | --- |
|  | **n=9** | **n=9** |  |
| **HR** | 549.33±42.6 | 553.33±71.22 | 0.8869 |
| **LVEDD (mm)** | 3.62±0.24 | 3.82±0.18 | 0.0800 |
| **LVESD (mm)** | 2.1±0.18 | 2.43±0.18 | 0.0024 |
| **PWTd (mm)** | 0.77±0.03 | 0.74±0.03 | 0.0274 |
| **PWTs (mm)** | 1.26±0.03 | 1.23±0.03 | 0.0043 |
| **FS%** | 41.98±1.92 | 36.38±3.15 | 0.0003 |
| **EF%** | 83.96±2.4 | 72.76±3.3 | 0.0004 |
| **r/h** | 2.34±0.21 | 2.56±0.18 | 0.0372 |

*Echocardiography Analysis at 5 weeks in the Pembrolizumab cohort. Values are presented as mean ± SD. HR: Heart Rate; LVEDD: Left Ventricular End Diastolic Diameter (mm); LVESD: Left Ventricular End Systolic Diameter (mm); PWTd: Posterior Wall Thickness diastole (mm); FS%: % Fractional Shortening, EF%: % Ejection Fraction; r/h: ratio of LV radius to PWT-posterior wall thickness. Student’s T-Test, unpaired, two-way.*

**Supplemental Table 4: Echocardiography analysis at 2 weeks in the Control, Pem +Atorv, IgG4+ Atorv and Pem groups**

| **Supplemental Table 4** | **Control (IgG4)** | **Pem** | **IgG4+Atorv** | **Pem +Atorv** |
| --- | --- | --- | --- | --- |
|  | **n=6** | **n=6** | **n=5** | **n=6** |
| **HR** | 503.66±67.23 | 575.00±25.33* | 539.20±43.41 | 596.88±62.22** |
| **LVEDD (mm)** | 3.76±0.24 | 3.87±0.24 | 3.55±1.62 | 3.90±0.39 |
| **LVESD (mm)** | 2.31±0.22 | 2.59±0.17*† | 2.25±0.15 | 2.52±0.29 |
| **PWTd (mm)** | 0.75±0.02 | 0.74±0.02 | 0.75±0.02 | 0.76±0.02 |
| **PWTs (mm)** | 1.22±3.43 | 1.21±0.02 | 1.21±0.02 | 1.23±0.02 |
| **FS%** | 38.75±3.43 | 32.93±3.53**† | 36.61±2.38 | 35.45±2.08 |
| **EF%** | 76.81±3.96 | 69.63±4.75**† | 74.46±2.79 | 73.04±2.60 |
| **r/h** | 2.51±0.20 | 2.60±0.22 | 2.35±0.2 | 2.55±0.32 |

*Echocardiography Analysis at 2 weeks in the Pembrolizumab + Atorvastatin cohort. Values are presented as mean ± SD. HR: Heart Rate; LVEDD: Left Ventricular End Diastolic Diameter (mm); LVESD: Left Ventricular End Systolic Diameter (mm); PWTd: Posterior Wall Thickness diastole (mm); FS%: % Fractional Shortening, EF%: % Ejection Fraction; r/h: ratio of LV radius to PWT-posterior wall thickness. *P<0.05, **P<0.01 vs control, †P<0.05 vs Atorv. One-Way ANOVA of Variance, Tukey’s post-hoc analysis.*

**Supplemental Table 5: Echocardiography analysis at 5 weeks in the Control, Pem +Atorv, IgG4+ Atorv and Pem groups**

| **Supplemental Table 5** | **Control (IgG4)** | **Pem** | **IgG4+Atorv** | **Pem +Atorv** |
| --- | --- | --- | --- | --- |
|  | **n=6** | **n=6** | **n=5** | **n=6** |
| **HR** | 563.66±64.18 | 569.50±28.51 | 531.40±42.18 | 565.33±50.88 |
| **LVEDD (mm)** | 3.58±0.42 | 3.99±0.32 | 3.74±0.39 | 3.72±0.24 |
| **LVESD (mm)** | 2.17±0.34 | 2.78±0.22**♯ | 2.45±0.32 | 2.32±0.32 |
| **PWTd (mm)** | 0.79±0.02 | 0.71±0.02***♯♯♯ | 0.73±0.02**♯♯ | 0.78±0.02 |
| **PWTs (mm)** | 1.26±0.02 | 1.22±0.02*♯ | 1.22±0.02*♯ | 1.26±0.02 |
| **FS%** | 39.45±3.55 | 30.28±1.64***†♯♯♯ | 34.51±3.42* | 37.71±4.46 |
| **EF%** | 77.61±1.633.99 | 66.07±2.42***†♯♯♯ | 71.77±4.21* | 75.52±5.02 |
| **r/h** | 2.27±0.37 | 2.80±0.20**♯ | 2.57±0.37 | 2.37±0.27 |

*Echocardiography Analysis at 5 weeks in the Pembrolizumab + Atorvastatin cohort. Values are presented as mean ± SD. HR: Heart Rate; LVEDD: Left Ventricular End Diastolic Diameter (mm); LVESD: Left Ventricular End Systolic Diameter (mm); PWTd: Posterior Wall Thickness diastole (mm); FS%: % Fractional Shortening, EF%: % Ejection Fraction; r/h: ratio of LV radius to PWT-posterior wall thickness. *P<0.05, **P<0.01, ***P<0.001 vs Control, †P<0.05 vs Atorv, ♯ P<0.05, ♯♯ P<0.01, ♯♯♯P<0.001 vs Pem+Atorv. One-Way ANOVA of Variance, Tukey’s post-hoc analysis.*

**Supplemental Table 6: RT-PCR Primers used in pAVCs and splenocytes**

| **Supplemental Table 6** | | | | |
| --- | --- | --- | --- | --- |
| Gene Name | Description | Forward Primer | Reverse Primer | Product Size |
| Atg5 | Autophagy related 5, Mus musculus | AAAGTCAAGTGATCAACGAAATGC | CGCTCCGTCGTGGTCATT | 80 |
| Becn2 | Beclin 2, Mus musculus | GGAAGAGGCTAACTCAGGAGAG | TTCTGTAGACATCATCCTGGCTGG | 97 |
| Bnip3 | Bnip3, Mus musculus | AAACAGCACTCTGTCTGAGGA | TCGACTTGACCAATCCCATATC | 100 |
| Canx | Calnexin, Mus musculus | TCTGGCAGCGACCTATGATT | TGGTTTCCAGATTCCCTGGTAG | 95 |
| Cd274 | CD274 antigen, Mus musculus | TCACTTGCTACGGGCGTTTA | GGGAATCTGCACTCCATCGT | 90 |
| Ctla4 | Cytotoxic T-lymphocyte-associated protein 4, Mus musculus | TCCCAGTCTTCTCTGAAGCCAT | TCACATGGAAAGCTGGCGAC | 85 |
| Ddit3 | DNA damage inducible transcript 3, Mus musculus | CCTGAGGAGAGAGTGTTCCAG | GACCAGGTTCTGCTTTCAGGT | 70 |
| Ifng | Interferon gamma, Mus musculus | ACTCAAGTGGCATAGATGTGGAA | TTTCATGTCACCATCCTTTTGCC | 80 |
| Il10 | Interleukin 10, Mus musculus | TGTCATCGATTTCTCCCCTGT | CACCTTGGTCTTGGAGCTTATT | 85 |
| Il17a | Interleukin 17α, Mus musculus | GGAGAGCTTCATCTGTGTCTCTG | TTTGAGGGATGATCGCTGCT | 84 |
| Il1b | Interleukin 1 beta, Mus musculus | TGCCACCTTTTGACAGTGATGA | GGTTTGGAAGCAGCCCTTCA | 80 |
| Il6 | Interleukin 6, Mus musculus | AGTCCTTCCTACCCCAATTTCC | TGGTCTTGGTCCTTAGCCAC | 80 |
| Il8 | Interleukin 8, Mus musculus | GCAACAGAAAGGAAGTGATAGCAG | GTAGCCTTCACCCATGGAGC | 82 |
| Inos (Nos2) | Nitric oxide synthase 2, Mus musculus | AGGCAATCTTCGTTCAGCCA | TAGCCCGCATAGCGTATCAG | 99 |
| Map1lc3a | Microtubule associated protein 1 light chain 3 alpha, Mus musculus | CACACCCATCGCTGACATCTA | AGGTTTCTTGGGAGGCGTAG | 80 |
| Map1lc3b | Μicrotubule associated protein 1 light chain 3 beta, Mus musculus | GGACCCTAACCCCATAGGAG | GCTCTATAATCACCCGCCTG | 95 |
| Padi4 | Peptidyl arginine deiminase, type IV, Mus musculus | AGAATGCCTATGTGGAGAGCTG | AGCTGCGGAATGTCGATGAT | 97 |
| Pdcd1 | Programmed cell death 1, Mus musculus | CAAGGACGACACTCTGAAGGA | TCTTCTCTCGTCCCTGGAAGT | 89 |
| Rela | RELA proto-oncogene, NF-kB subunit, Mus musculus | CCTCTGGCGAATGGCTTTAC | TGAGGGGAAACAGATCGTCC | 97 |
| Tgfb1 | Transforming growth factor beta 1, Mus musculus | GATACGCCTGAGTGGCTGTC | AAGCCCTGTATTCCGTCTCC | 80 |
| Tnf | Tumor necrosis factor, Mus musculus | ATGGCCTCCCTCTCATCAGT | TGGTTTGCTACGACGTGGG | 100 |

*Primers designed for RT-PCR analysis of the ICIs effect on pAVCs and splenocytes in vitro*

**Supplemental Table 7: Primers designed for PCR amplification of human and murine PDCD1/** **Pdcd1 extracellular domains.**

| Supplemental Table 7 | | | | |
| --- | --- | --- | --- | --- |
| Primers | Forward primer | Reverse primer | Product Size | Tm (°C) |
| PDCD1  (*Homo sapiens*) | GGTAATTGCATATGTTAGACTCCCCAGACAGGCCCT | CCGGAATTCTCATTGGAACTGGCCGGCTGGCCT | 455 | 68°C/74°C |
| Pdcd1  (*Mus musculus*) | GGTAATTGCATATGCTAGAGGTCCCCAATGGGCCCT | CCGGAATTCTCATTGAAACCGGCCTTCTGGTTT | 455 | 70°C/67°C |

*Primers designed for PCR amplification of human PDCD1 and murine Pdcd1 extracellular domain.*

**Supplemental Figure Legends**

**Supplemental Fig. 1: Workflows of the *in vitro* and *in vivo* experiments.** Representative workflows of **a.** *In* *vitro* experiments on pAVCs and splenocytes **b.** *In vivo* model of Pembrolizumab-induced cardiotoxicity **c.** *In vitro* human-based experiments on PBMCs and EAHy-926 cells **d.** *In vivo* model of atorvastatin prophylaxis against Pembrolizumab-induced cardiotoxicity. *Atorv: Atorvastatin, pAVCs: Primary adult ventricular cardiomyocytes, Ave: Avelumab, Ipi: Ipilimumab, PBMCs: Peripheral blood mononuclear cells, Pem: Pembrolizumab, Prav: Pravastatin.*

**Supplemental Fig. 2: Only Pembrolizumab induced immune-cell mediated toxicity in primary AVCs. a.** Graph of cellular viability assessed by MTT assay in primary splenocytes treated with Pem, Ipi and Ave (0-100μg/ml) for 24h (n=6/group). **b.** Heatmap of inflammatory gene expression in primary splenocytes treated with Pem, Ipi and Ave (0-100μg/ml) for 24 h (n=5-6/group). **c.** Graph of cellular viability assessed by MTT assay in primary AVCs treated with Pem, and Ave (0-100μg/ml) for 18h (n=5-6/group) **d.** Graph of cellular viability assessed by MTT assay in primary AVCs treated with Pem, and Ave conditioned media from primary splenocytes (0-100μg/ml) for 18h (n=6/group). Heatmaps of **e.** Inflammatory and **f.** Autophagy, ER-Stress and Apoptosis genes in primary AVCs treated with Pem conditioned media from primary splenocytes (n=6/group). Data are presented as mean ± SD. *P<0.05, **P<0.01, ****P<0.001, One-way or Two-way ANOVA of Variance, Tukey’s post-hoc analysis. *Atg5: Autophagy Related 5, Becn2: Beclin 2, Bnip: BCL2 Interacting Protein 2, Canx: Calnexin, Ddit3: DNA damage inducible transcript 3, Il: Interleukin, Lc3b/a: Microtubule Associated Protein 1 Light Chain 3 Beta/alpha, Rela: nuclear factor NF-kappa-B p65 subunit, Tgfβ: Transforming growth factor β, Tnfα: Tumor necrosis factor α.*

**Supplemental Fig. 3: IgG4 isotype control does not lead to conformational changes and does not bind to human and murine PD-1-ED.** Representative circular dichroism (CD) spectra of **a.** murine and **b.** human PD-1-ED incubated with IgG4, isotype control, at 15^o^C at different timepoints.

**Supplemental Fig. 4:** **Gating strategy for the evaluation of immune subsets in the myocardium and the whole blood.** Total events acquired were first gated to exclude debris and doublets, and singlets were plotted on a CD11b/SSC dot plot. CD11b^+^ cells were further analyzed on a Ly6G/Ly6C dot plot for the discrimination of monocytes and neutrophils, whereas the CD11b^-^ compartment was plotted on a CD3/CD19 dot plot for the discrimination of T-cells and - cells, respectively. T cells were further analyzed on a CD8/CD4 dot plot for the discrimination of CD8^+^ and CD4^+^ T cells.

**Supplemental Fig. 5:** **Assessment of cardiac damage biomarkers after serial administration of IgG4 and Pem.** Graphs of **a.** cardiac Troponin I (pg/mL), **b.** Lactate dehydrogenase (U/L) and **c.** Creatine phosphokinase-MB (U/L) in the IgG4- and Pem-treated controls throughout the in vivo protocol. Data are presented as mean ± SD. *P<0.05 vs Baseline and †P<0.05 and ††P<0.01 vs Control. Two-way ANOVA of Variance, Tukey’s post-hoc analysis. *cTnI: cardiac Troponin I, CK-MB: Creatine phosphokinase-MB, DT: detection limit, LDH: Lactate dehydrogenase*.

**Supplemental Fig. 6: Pembrolizumab induces concomitant CD4^+^ and CD8^+^ T-cell increase at 5 weeks of administration.** Graph of the flow cytometry subpopulation analysis of the whole blood at 5 weeks of IgG4 or Pem administration for **a.** T- **b.** CD4 T- **c.** CD8 T- **d.** B and **e**. NK-cells expressed as % ratio of parent sub-population (n=8-9/group). Data are presented as mean ± SD. *P<0.05, ****P<0.001, Two-way ANOVA of Variance, Tukey’s post-hoc analysis.

**Supplemental Figures**

**
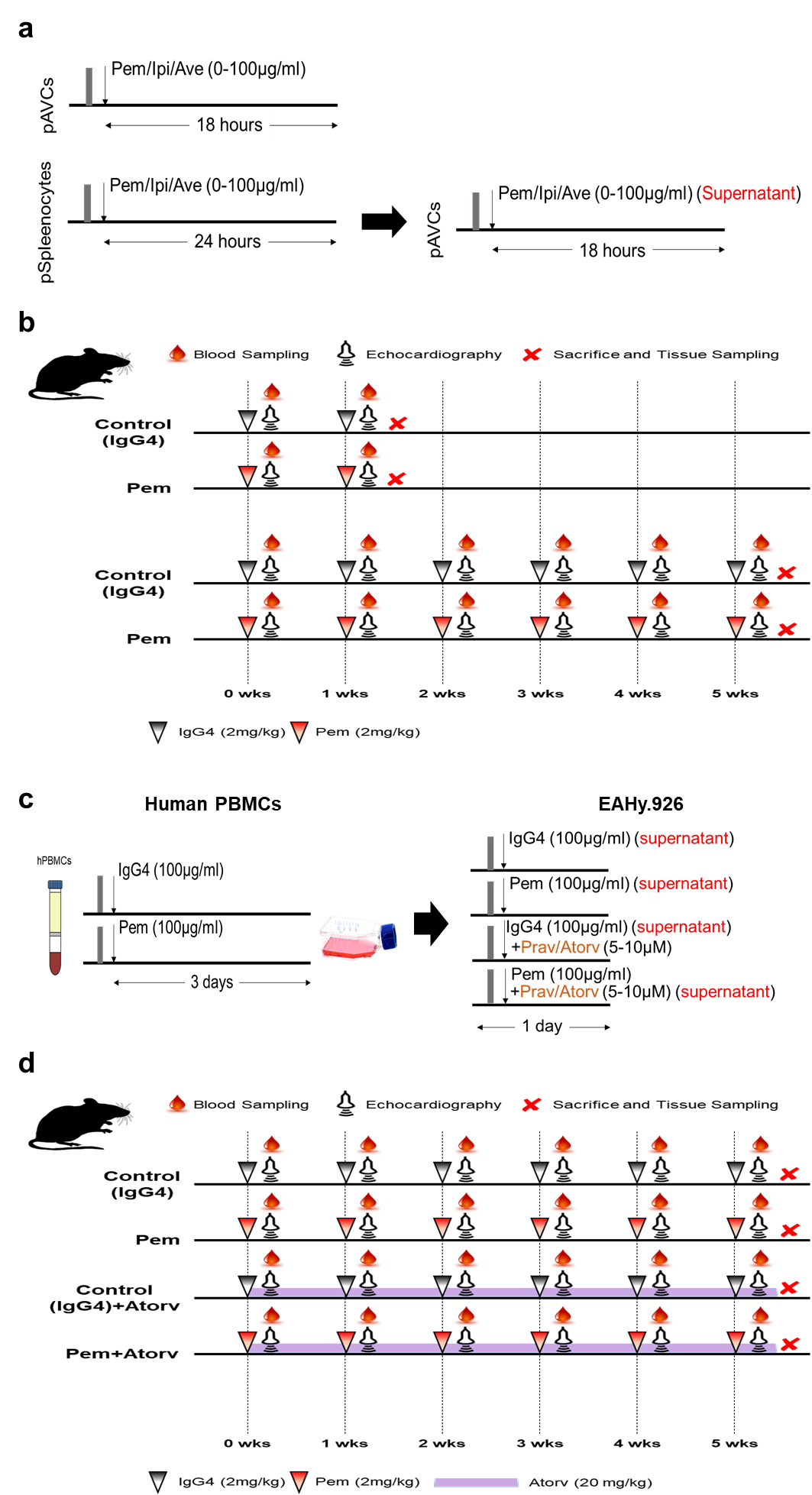
**

**Supplemental Fig. 1**


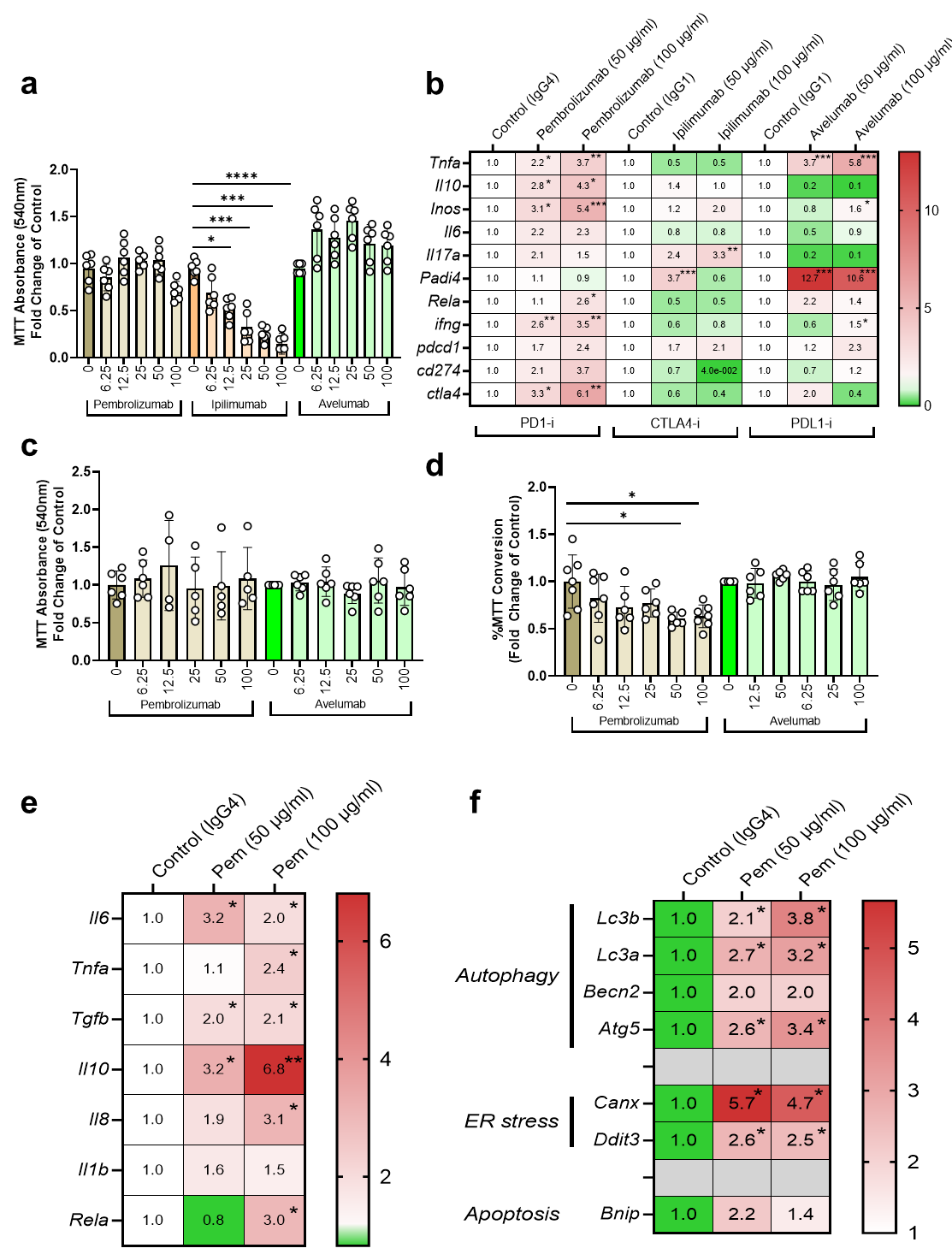


**Supplemental Fig. 2**

**
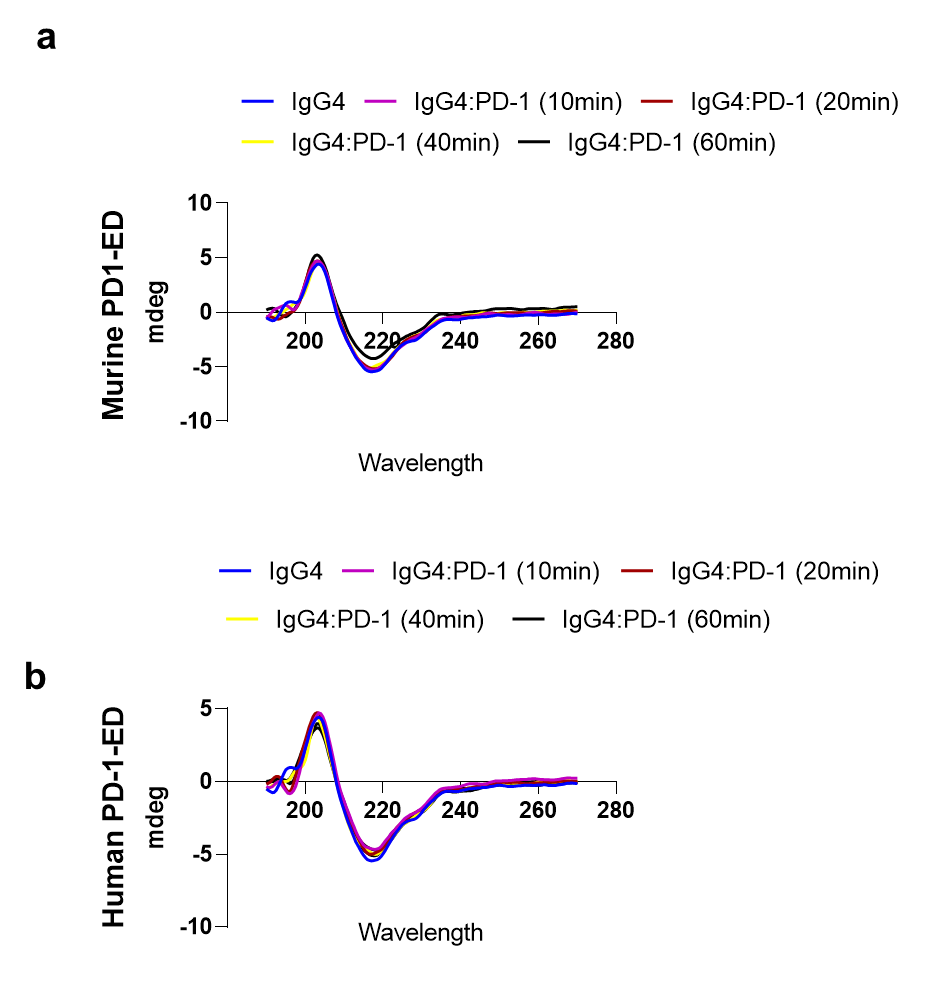
**

**Supplemental Fig. 3**

**
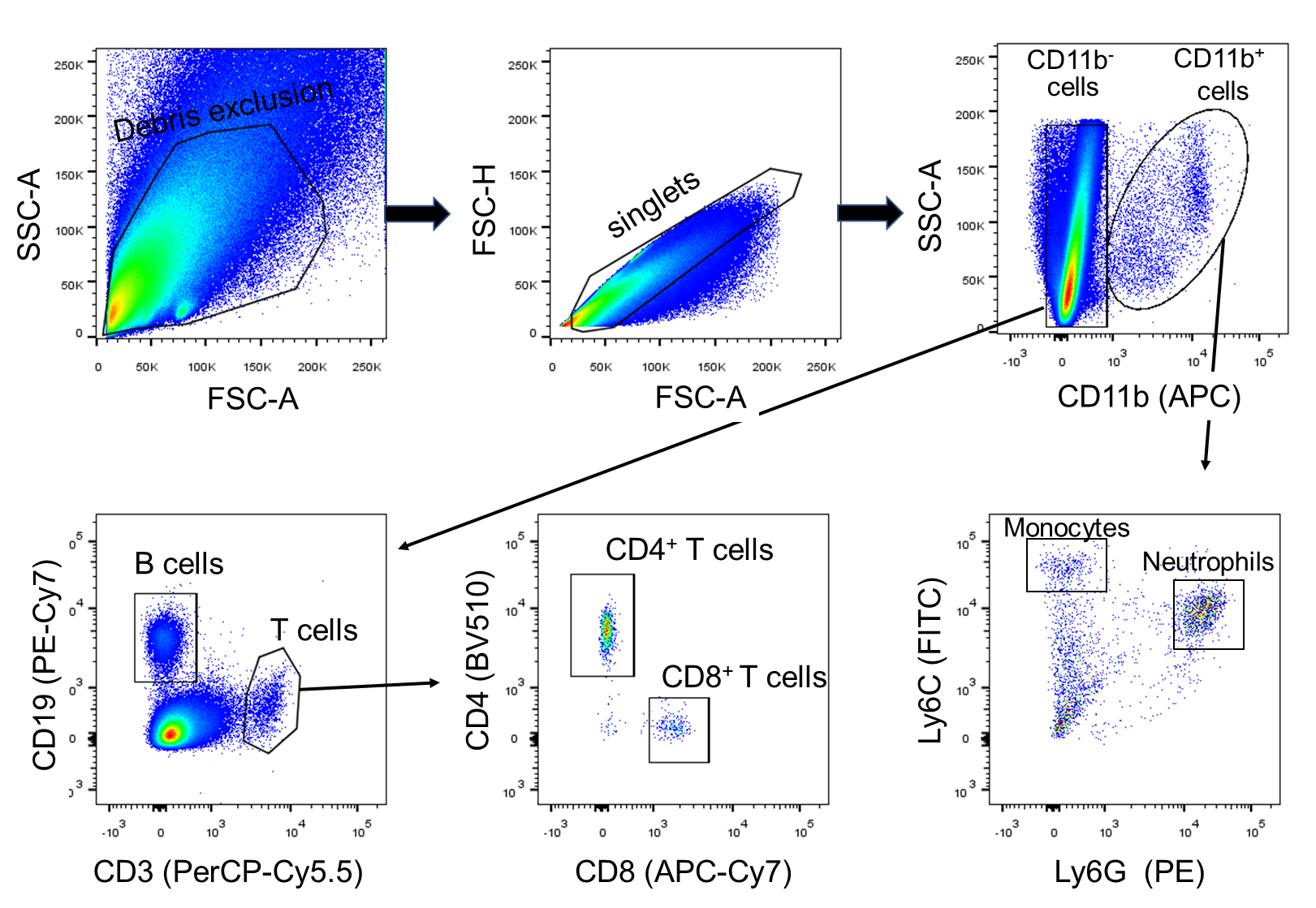
**

**Supplemental Fig. 4**

**Supplemental Fig. 5**


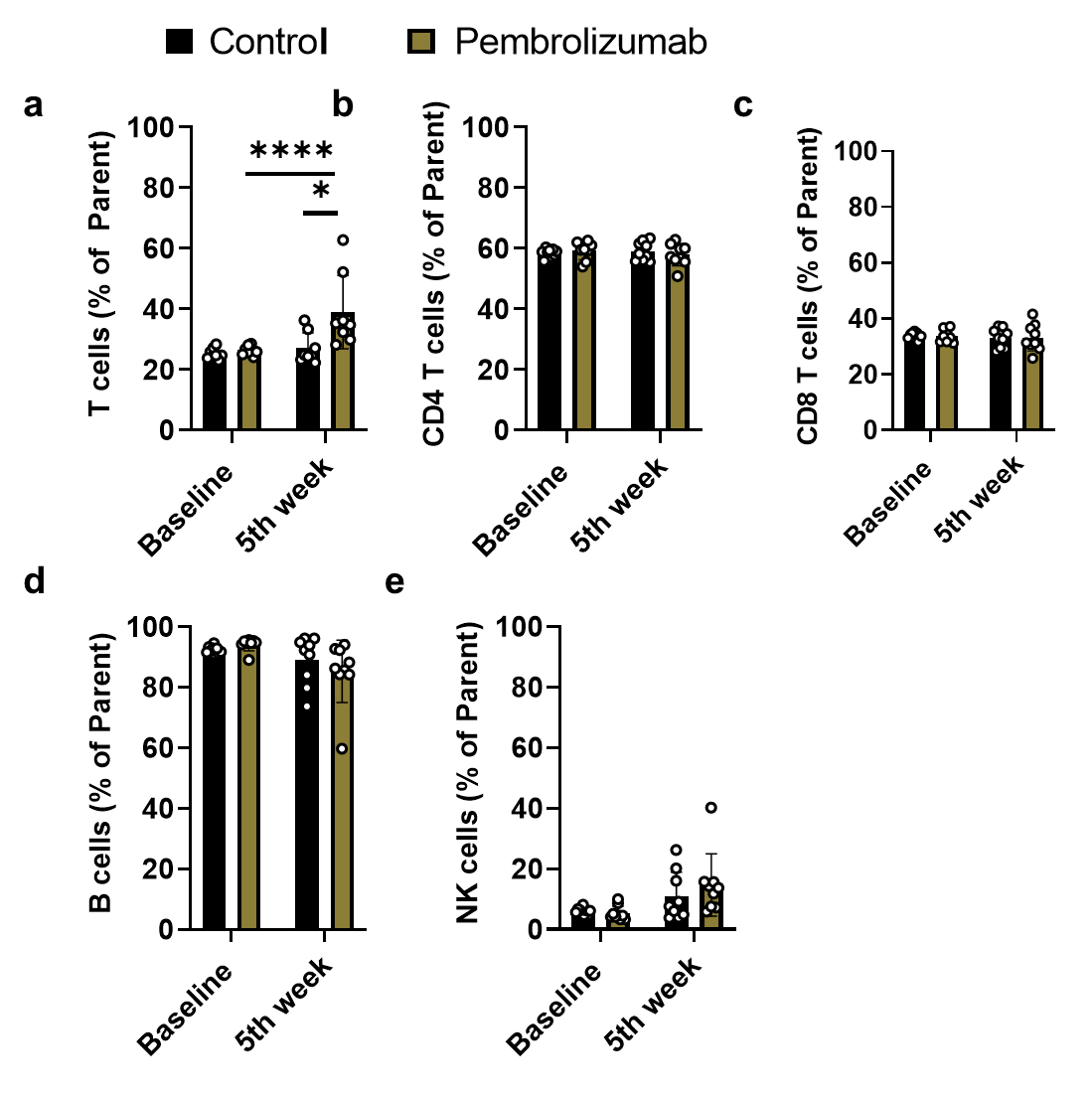


**Supplemental Fig. 6**

**References**

1. Baaten BJ, Tinoco R, Chen AT, Bradley LM (2012) Regulation of Antigen-Experienced T Cells: Lessons from the Quintessential Memory Marker CD44. Front Immunol 3:23 doi:10.3389/fimmu.2012.00023

2. Colovai AI, Giatzikis C, Ho EK, Farooqi M, Suciu-Foca N, Cattoretti G, Orazi A (2004) Flow cytometric analysis of normal and reactive spleen. Mod Pathol 17:918-927 doi:10.1038/modpathol.3800141

3. Dymkowska D, Wrzosek A, Zablocki K (2021) Atorvastatin and pravastatin stimulate nitric oxide and reactive oxygen species generation, affect mitochondrial network architecture and elevate nicotinamide N-methyltransferase level in endothelial cells. J Appl Toxicol 41:1076-1088 doi:10.1002/jat.4094

4. Efentakis P, Kremastiotis G, Varela A, Nikolaou PE, Papanagnou ED, Davos CH, Tsoumani M, Agrogiannis G, Konstantinidou A, Kastritis E, Kanaki Z, Iliodromitis EK, Klinakis A, Dimopoulos MA, Trougakos IP, Andreadou I, Terpos E (2019) Molecular mechanisms of carfilzomib-induced cardiotoxicity in mice and the emerging cardioprotective role of metformin. Blood 133:710-723 doi:10.1182/blood-2018-06-858415

5. Efentakis P, Lamprou S, Makridakis M, Barla I, Nikolaou PE, Christodoulou A, Dimitriou C, Kostomitsopoulos N, Ntanasis-Stathopoulos I, Theochari I, Gavriatopoulou M, Gakiopoulou H, Tasouli A, Vlahou A, Gikas E, Thomaidis N, Dimopoulos MA, Terpos E, Andreadou I (2022) Mineralocorticoid Receptor Pathway Is a Key Mediator of Carfilzomib-induced Nephrotoxicity: Preventive Role of Eplerenone. Hemasphere 6:e791 doi:10.1097/HS9.0000000000000791

6. Efentakis P, Varela A, Chavdoula E, Sigala F, Sanoudou D, Tenta R, Gioti K, Kostomitsopoulos N, Papapetropoulos A, Tasouli A, Farmakis D, Davos CH, Klinakis A, Suter T, Cokkinos DV, Iliodromitis EK, Wenzel P, Andreadou I (2020) Levosimendan prevents doxorubicin-induced cardiotoxicity in time- and dose-dependent manner: implications for inotropy. Cardiovasc Res 116:576-591 doi:10.1093/cvr/cvz163

7. Elassaiss-Schaap J, Rossenu S, Lindauer A, Kang SP, de Greef R, Sachs JR, de Alwis DP (2017) Using Model-Based "Learn and Confirm" to Reveal the Pharmacokinetics-Pharmacodynamics Relationship of Pembrolizumab in the KEYNOTE-001 Trial. CPT Pharmacometrics Syst Pharmacol 6:21-28 doi:10.1002/psp4.12132

8. Greenfield NJ (2006) Using circular dichroism spectra to estimate protein secondary structure. Nat Protoc 1:2876-2890 doi:10.1038/nprot.2006.202

9. Heiberg E, Sjogren J, Ugander M, Carlsson M, Engblom H, Arheden H (2010) Design and validation of Segment--freely available software for cardiovascular image analysis. BMC Med Imaging 10:1 doi:10.1186/1471-2342-10-1

10. Horinouchi H, Yamamoto N, Fujiwara Y, Sekine I, Nokihara H, Kubota K, Kanda S, Yagishita S, Wakui H, Kitazono S, Mizugaki H, Tokudome T, Tamura T (2015) Phase I study of ipilimumab in phased combination with paclitaxel and carboplatin in Japanese patients with non-small-cell lung cancer. Invest New Drugs 33:881-889 doi:10.1007/s10637-015-0243-5

11. Jin H, D'Urso V, Neuteboom B, McKenna SD, Schweickhardt R, Gross AW, Fomekong Nanfack Y, Paoletti A, Carter C, Toleikis L, Fluck M, Scheuenpflug J, Cai T (2021) Avelumab internalization by human circulating immune cells is mediated by both Fc gamma receptor and PD-L1 binding. Oncoimmunology 10:1958590 doi:10.1080/2162402X.2021.1958590

12. Jonkman J, Brown CM, Wright GD, Anderson KI, North AJ (2020) Tutorial: guidance for quantitative confocal microscopy. Nat Protoc 15:1585-1611 doi:10.1038/s41596-020-0313-9

13. Kozakov D, Hall DR, Xia B, Porter KA, Padhorny D, Yueh C, Beglov D, Vajda S (2017) The ClusPro web server for protein-protein docking. Nat Protoc 12:255-278 doi:10.1038/nprot.2016.169

14. Kwiatkowski G, Bar A, Jasztal A, Chlopicki S (2021) MRI-based in vivo detection of coronary microvascular dysfunction before alterations in cardiac function induced by short-term high-fat diet in mice. Sci Rep 11:18915 doi:10.1038/s41598-021-98401-1

15. Lee JY, Lee HT, Shin W, Chae J, Choi J, Kim SH, Lim H, Won Heo T, Park KY, Lee YJ, Ryu SE, Son JY, Lee JU, Heo YS (2016) Structural basis of checkpoint blockade by monoclonal antibodies in cancer immunotherapy. Nat Commun 7:13354 doi:10.1038/ncomms13354

16. Lessard JC (2013) Molecular cloning. Methods Enzymol 529:85-98 doi:10.1016/B978-0-12-418687-3.00007-0

17. Micsonai A, Wien F, Bulyaki E, Kun J, Moussong E, Lee YH, Goto Y, Refregiers M, Kardos J (2018) BeStSel: a web server for accurate protein secondary structure prediction and fold recognition from the circular dichroism spectra. Nucleic Acids Res 46:W315-W322 doi:10.1093/nar/gky497

18. Richaud AD, Zaghouani M, Zhao G, Wangpaichitr M, Savaraj N, Roche SP (2022) Exploiting the Innate Plasticity of the Programmed Cell Death-1 (PD1) Receptor to Design Pembrolizumab H3 Loop Mimics. Chembiochem 23:e202200449 doi:10.1002/cbic.202200449

19. Rosano GL, Ceccarelli EA (2014) Recombinant protein expression in Escherichia coli: advances and challenges. Front Microbiol 5:172 doi:10.3389/fmicb.2014.00172

20. Scapin G, Yang X, Prosise WW, McCoy M, Reichert P, Johnston JM, Kashi RS, Strickland C (2015) Structure of full-length human anti-PD1 therapeutic IgG4 antibody pembrolizumab. Nat Struct Mol Biol 22:953-958 doi:10.1038/nsmb.3129

21. Schuler R, Efentakis P, Wild J, Lagrange J, Garlapati V, Molitor M, Kossmann S, Oelze M, Stamm P, Li H, Schafer K, Munzel T, Daiber A, Waisman A, Wenzel P, Karbach SH (2019) T Cell-Derived IL-17A Induces Vascular Dysfunction via Perivascular Fibrosis Formation and Dysregulation of (.)NO/cGMP Signaling. Oxid Med Cell Longev 2019:6721531 doi:10.1155/2019/6721531

22. Stuckey DJ, Carr CA, Tyler DJ, Aasum E, Clarke K (2008) Novel MRI method to detect altered left ventricular ejection and filling patterns in rodent models of disease. Magn Reson Med 60:582-587 doi:10.1002/mrm.21677

23. Tsoumani M, Georgoulis A, Nikolaou PE, Kostopoulos IV, Dermintzoglou T, Papatheodorou I, Zoga A, Efentakis P, Konstantinou M, Gikas E, Kostomitsopoulos N, Papapetropoulos A, Lazou A, Skaltsounis AL, Hausenloy DJ, Tsitsilonis O, Tseti I, Di Lisa F, Iliodromitis EK, Andreadou I (2021) Acute administration of the olive constituent, oleuropein, combined with ischemic postconditioning increases myocardial protection by modulating oxidative defense. Free Radic Biol Med 166:18-32 doi:10.1016/j.freeradbiomed.2021.02.011

24. Tyrankiewicz U, Skorka T, Orzylowska A, Jablonska M, Jasinski K, Jasztal A, Bar A, Kostogrys R, Chlopicki S (2016) Comprehensive MRI for the detection of subtle alterations in diastolic cardiac function in apoE/LDLR(-/-) mice with advanced atherosclerosis. NMR Biomed 29:833-840 doi:10.1002/nbm.3524

25. Wang Y, Zhang K, Georgiev P, Wells S, Xu H, Lacey BM, Xu Z, Laskey J, McLeod R, Methot JL, Bittinger M, Pasternak A, Ranganath S (2020) Pharmacological inhibition of hematopoietic progenitor kinase 1 positively regulates T-cell function. PLoS One 15:e0243145 doi:10.1371/journal.pone.0243145

26. Xu L, Liu Y, He X (2006) Expression and purification of soluble human programmed death-1 in Escherichia coli. Cell Mol Immunol 3:139-143

27. Yamaguchi H, Miyazaki M (2014) Refolding techniques for recovering biologically active recombinant proteins from inclusion bodies. Biomolecules 4:235-251 doi:10.3390/biom4010235

28. Yu F, Fu R, Liu L, Wang X, Wu T, Shen W, Gui Z, Mo X, Fang B, Xia L (2019) Leptin-Induced Angiogenesis of EA.Hy926 Endothelial Cells via the Akt and Wnt Signaling Pathways In Vitro and In Vivo. Front Pharmacol 10:1275 doi:10.3389/fphar.2019.01275

29. Zhang X, Schwartz JC, Guo X, Bhatia S, Cao E, Lorenz M, Cammer M, Chen L, Zhang ZY, Edidin MA, Nathenson SG, Almo SC (2004) Structural and functional analysis of the costimulatory receptor programmed death-1. Immunity 20:337-347 doi:10.1016/s1074-7613(04)00051-2

30. Zhansaya A, Kanatbek M, Kanat T, Bakhytkali I, Darkhan K, Arman K, Pavel T, Kasym M, Yerlan R (2020) Recombinant Expression and Purification of Extracellular Domain of the Programmed Cell Death Protein Receptor. Rep Biochem Mol Biol 8:347-357
